# Predicting Long‐Term Depression Progression in Parkinson's Disease: A Machine‐Learning Survival Analysis and Risk Score

**DOI:** 10.1002/cns.70845

**Published:** 2026-03-28

**Authors:** Defu Liu, Chong Qi, Jiansong Huang, Hutao Xie, Yutong Zhuang, Quan Zhang, Xuemin Zhao, Tianqi Hu, Guofan Qin, Yue Lu, Xin Zhang, Sizhe Li, Mingyu Yang, Jianguo Zhang, Yin Jiang

**Affiliations:** ^1^ Department of Neurosurgery, Beijing Tiantan Hospital Capital Medical University Beijing China; ^2^ Department of Clinical Medicine Peking University Health Science Center Beijing China; ^3^ Department of Multimodal Neuroimaging and Brain Function, Beijing Neurosurgical Institute Beijing Tiantan Hospital, Capital Medical University Beijing China; ^4^ Department of Functional Neurosurgery Beijing Neurosurgical Institute, Capital Medical University Beijing China; ^5^ Department of Neurophysiology Beijing Neurosurgical Institute, Capital Medical University Beijing China; ^6^ School of Basic Medical Sciences Capital Medical University Beijing China

**Keywords:** depression, machine learning, Parkinson's disease, risk stratification, survival analysis

## Abstract

**Background:**

Depression in Parkinson's disease (dPD) is common and heterogeneous, impairs quality of life, and may accelerate disease progression. Tools that predict long‐term dPD progression are lacking.

**Methods:**

We retrospectively analyzed de novo, drug‐naïve Parkinson's disease (PD) participants in the Parkinson's Progression Markers Initiative (PPMI; 2011–2024). The primary outcome was depressive progression, defined as a sustained worsening in Geriatric Depression Scale‐15 (GDS‐15) category over 12 months. Candidate predictors included demographic, motor, and non‐motor variables at both total and sub‐item levels. Four survival machine learning models, Random Survival Forests (RSF), Extreme Gradient Boosting, Support Vector Survival Machines, and Gradient Boosting Survival Analysis, were evaluated using concordance index (C‐index). Shapley Additive exPlanations were applied to identify key predictors and construct an integer‐based risk score.

**Results:**

Of 1819 eligible participants, 496 met inclusion criteria (median age 62 years [IQR: 55–69]; 61.3% male); 94 (19.0%) progressed over a median 6 year follow‐up. RSF achieved the best discrimination (test‐set C‐index 0.744). Key predictors included age, baseline GDS‐15; SCOPA–AUT subscores (thermoregulatory, gastrointestinal, cardiovascular); cognition (BJLOT, SDMT); impulse control disorder (QUIP–CS score), and MDS–UPDRS I (sleep problems night, pain and other sensations). The SHAP‐derived score stratified patients into low (progression 7.3%), moderate (14.7%), and high‐risk (36.5%) groups with clear Kaplan–Meier separation (log‐rank *p* < 0.001). Time‐dependent AUCs were 0.721, 0.770, 0.794, 0.792, and 0.812 at 2, 4, 6, 8, and 10 years.

**Conclusions:**

An explainable survival model and integer‐based risk score using routinely collected measures predicted long‐term dPD progression and enabled pragmatic risk stratification to support early, personalized management.

## Graphical abstract

We developed and internally validated a machine learning–based survival model for predicting long‐term progression of dPD. Supported by SHAP analysis, the model identified that depressive symptom severity, cognitive impairment, and autonomic dysfunction were the main key clinical predictors for dPD progression. We found motor symptoms contribute minimally to predicting dPD progression. Utilizing the model, we stratified patients into low, moderate, and high‐risk groups, guiding early intervention and personalized management.

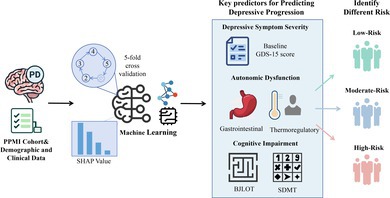

**Abbreviations**
AUC: area under the curve
BJLOT: Benton judgment of line orientation test
C‐index: concordance index
dPD: depression in Parkinson's disease
ESS: Epworth sleepiness scale
GBSA: gradient boosting survival analysis
GDS‐15: geriatric depression scale‐short form
HVLT‐R: Hopkins verbal learning test‐revised
LNS: letter‐number sequencing test
MDS‐UPDRS: movement disorder society revision of the unified parkinson disease rating scale
MSEADL: modified schwab and england activities of daily living
MSFT: modified semantic fluency test
PD: Parkinson's disease
PDD: persistent depressive disorder
PPMI: Parkinson's progression markers initiative
QUIP‐CS: questionnaire for impulsive‐compulsive disorders in Parkinson's disease–current short version
RBDSQ: rapid eye movement sleep behavior disorder screening questionnaire
RSF: random survival forests
SCOPA‐AUT: scales for outcomes in Parkinson's disease‐Autonomic questionnaire
SDMT: symbol digit modalities test
SHAP: Shapley additive explanations
SLF‐III: superior longitudinal fasciculus III
SSVM: support vector survival machines
STAI: state trait anxiety total score
time‐ROC: time‐dependent receiver operating characteristic
XGBoost: extreme gradient boosting

## 1 Introduction

Depression in Parkinson's disease (dPD) is common (> 40%) and a major driver of disability and poor quality of life [1, 2, 3]. However, its natural history is highly heterogeneous: dPD can emerge at any stage–including prodromally before motor onset–and individual trajectories differ, with some patients showing progressive worsening while others stabilize or partially remit [4, 5]. This variability hampers one–size–fits–all management. Accordingly, an individualized model that predicts whether and how dPD will progress over time is needed to identify high‐risk patients early and support more informed clinical decisions.

Multiple studies have identified reliable biomarkers for predicting depression development in PD, including earlier age at onset, longer disease duration, autonomic dysfunction, and higher UPDRS motor scores [6, 7]. However, prior models primarily used dichotomous classification to predict depression occurrence and failed to incorporate time–to–event data [7, 8], limiting their ability to track individual risk trajectories over time. Furthermore, conventional approaches often rely on composite scores (e.g., total UPDRS‐III or global autonomic scales), which may obscure the predictive value of specific sub–items. These limitations highlight the need for more sophisticated modeling approaches that incorporate time–to–event data and granular clinical features.

The Parkinson's Progression Markers Initiative (PPMI) is an ongoing, multicenter, prospective observational study focusing on early‐stage, drug‐naïve PD patients [9]. It incorporates comprehensive assessments, including depression scales, motor and non–motor symptoms, cognitive function, and autonomic dysfunction. Due to its rigorous design and longitudinal follow‐up, the PPMI dataset is well‐suited for modeling dPD progression.

Leveraging PPMI, we aimed to develop and internally validate a survival machine‐learning model that predicts long‐term dPD progression and to derive a clinically applicable, interpretable risk score. We trained time–to–event algorithms on granular, subitem‐level clinical features and used Shapley Additive exPlanations (SHAP) to identify influential predictors and translate model importance into an integer‐based score with actionable thresholds, assessing discrimination and risk stratification using the concordance index, time‐dependent AUC, Kaplan–Meier curves, and log‐rank testing.

## 2 Methods

### 2.1 Study Design and Participants

**FIGURE 1:**
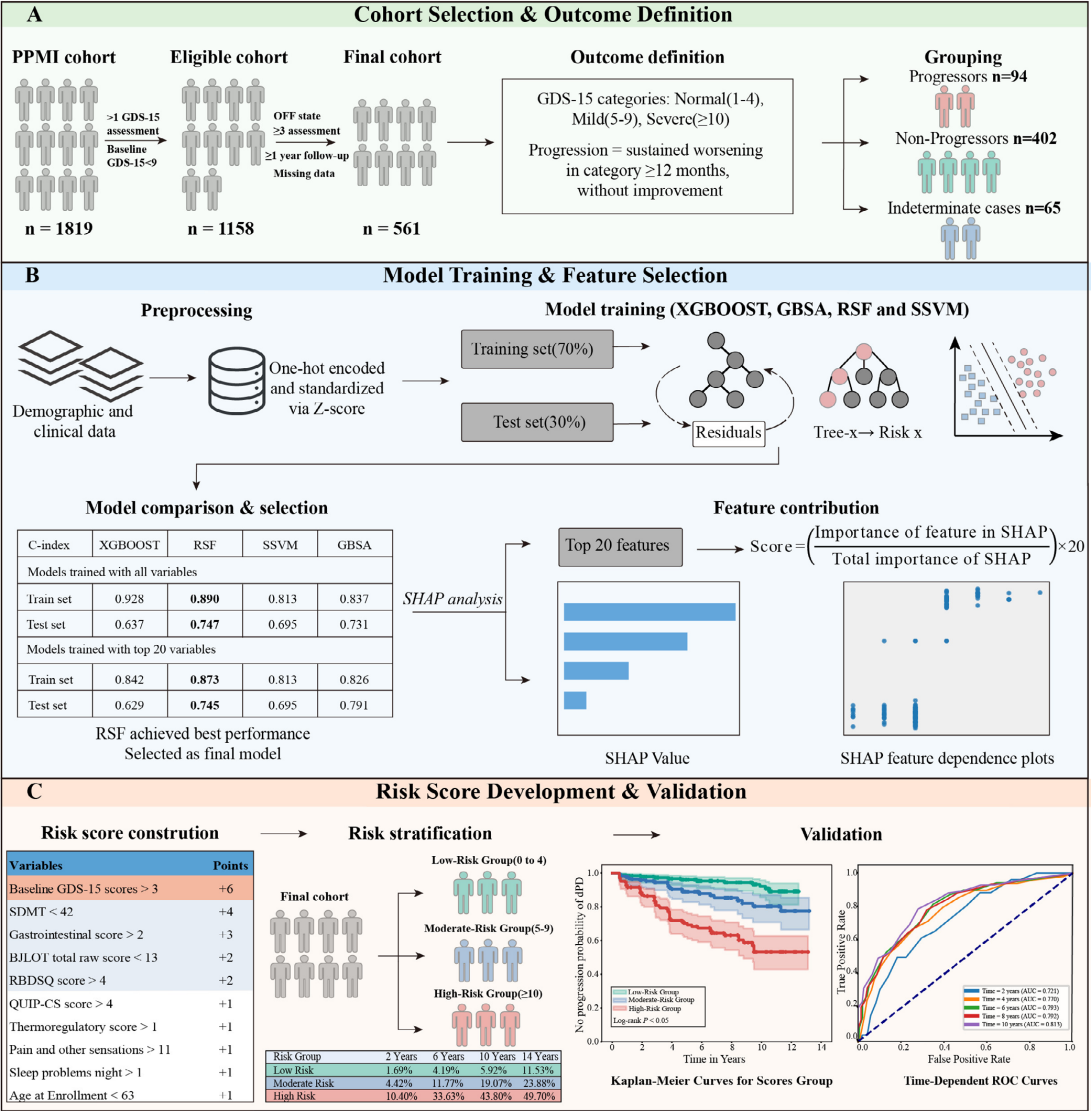
Flow diagram describing study workflow. C‐index, concordance index; GBSA, Gradient Boosting Survival Analysis; RSF, Random Survival Forest; SHAP, Shapley Additive exPlanations; SSVM, Survival Support Vector Machine; XGBoost, Extreme Gradient Boosting.

This retrospective prognostic modeling study (Figure 1) utilized prospectively collected data from the PPMI cohort, accessed on October 23, 2024 (www.ppmi‐info.org/data). PPMI is an ongoing prospective multicenter observational study designed to collect longitudinal data from approximately 4000 participants across 50 global sites [9]. Participating PPMI sites all received approval from an ethical standards committee before study initiation and written informed consent was obtained for all individuals participating in the study. The data that support the findings of this study are openly available in PPMI at https://www.ppmi‐info.org/access‐data‐specimens/download‐data, reference number NCT04477785. The authors confirm that the ethical policies of the journal, as noted on the journal's author guidelines page, have been adhered to. The study enrolled de novo, drug–naïve PD patients and conducted standardized longitudinal assessments. Detailed study protocols and data access information are available at www.ppmi‐info.org.

Participants were selected from the de novo PD cohort, defined as having a PD diagnosis within 2 years and being medication–naïve for motor symptoms at baseline (*n* = 1819). For this analysis, inclusion criteria were: (1) completion of baseline Geriatric Depression Scale‐Short Form (GDS–15) assessment, a validated instrument demonstrating robust properties in PD [10]; (2) at least three follow‐up assessments over a minimum period of 1 year; and (3) availability of complete data for the predictor variables, including demographic, motor, and non–motor measures. Participants were excluded if they had (1) a baseline GDS‐15 score > 9, indicative of severe depression; (2) comorbid psychiatric or neurological disorders potentially confounding depressive symptoms; or (3) insufficient follow‐up or missing data required to determine depressive progression status.

### 2.2 Outcome Definition

Previous studies frequently defined dPD using a GDS‐15 cutoff score ≥ 5 [7], which distinguished depression from non‐depression but did not differentiate severity levels. Furthermore, current diagnostic criteria for depression required a minimum symptom duration of 2 weeks for a severe depressive episode and 2 years for persistent depressive disorder (PDD) [11, 12]. Therefore, GDS‐15 scores were categorized into three severity levels based on validated cut‐off [10, 11, 12, 13, 14]: normal (1–4), mild depression (5–9), and severe depression (≥ 10). To ensure robust classification and minimize transient mood fluctuations, depressive progression was defined as a sustained categorical worsening (from normal to mild/severe or mild to severe depression), maintained for at least 12 consecutive months without subsequent improvement (from severe to mild/normal or mild to normal). Participants were classified into two groups: (1) Progressors, exhibiting sustained depressive worsening over 12 months; and (2) Non‐progressors, who remained stable or demonstrated improvement over the follow‐up period [11, 12]. Participants with depressive worsening but without sufficient follow–up to confirm sustained progression (i.e., < 12 months post‐transition) were classified as indeterminate and excluded.

### 2.3 Candidate Predictors

Candidate predictors were selected based on clinical expertise, empirical evidence from prior studies on the progression of dPD and accessibility [6, 7, 8, 9, 10, 11, 12, 13, 14, 15, 16]. These features included demographic, motor and non‐motor variables. Demographic factors encompassed age, sex, educational attainment, handedness, laterality of symptom onset, disease duration and depression history. GDS‐15 scale has been validated in non‐elderly and elderly PD patients with good accuracy for identifying major and non‐major depression in PD. Ability of daily activities was evaluated by Modified Schwab and England Activities of Daily Living (MSEADL). Cognitive function was evaluated by Benton Judgment of Line Orientation Test (BJLOT), Modified Semantic Fluency Test (MSFT), Symbol Digit Modalities Test (SDMT), Hopkins Verbal Learning Test‐Revised (HVLT‐R) and Letter‐Number Sequencing Test (LNS). Impulse control was evaluated by Questionnaire for Impulsive‐Compulsive Disorders in Parkinson's Disease‐Current Short Version (QUIP‐CS). Autonomic nervous function was evaluated by Scales for Outcomes in Parkinson's Disease‐Autonomic questionnaire (SCOPA‐AUT). Emotion state was evaluated by State Trait Anxiety Total Score (STAI). Sleep quality metrics was evaluated by Epworth Sleepiness Scale (ESS) and Rapid Eye Movement Sleep Behavior Disorder Screening Questionnaire (RBDSQ). Movement Disorder Society Revision of the Unified Parkinson Disease Rating Scale (MDS‐UPDRS) was utilized for non‐motor experiences of daily living (MDS‐UPDRS I), motor experiences of daily living (MDS‐UPDRS II) and motor examination (MDS‐UPDRS III). Specially, some sub–item manifestations in the scales were included and the details of candidate predictors were provided in the Table S1.

### 2.4 Model Development and Feature Selection

Continuous variables were standardized via Z‐score transformation, categorical variables were one‐hot encoded. Participants with missing data were excluded to maintain data integrity. To ensure the stability of the predictive model and mitigate overfitting, we a adopted rigorous internal validation strategy. A 5‐fold cross‐validation was implemented exclusively within the 70% training set to optimize hyperparameters using the “Optuna” framework. The final model was then fixed and evaluated on the independent 30% test set, which remained entirely unseen during the training and hyperparameter tuning phases. This approach provides a rigorous internal validation of the model's performance. Moreover, the training (70%) and testing (30%) subsets was stratified by event status and time‐to‐event variables to ensure balanced distribution. Four distinct machine learning survival models were trained: (1) Extreme Gradient Boosting (XGBoost), (2) Random Survival Forests (RSF), (3) Support Vector Survival Machines (SSVM), and (4) Gradient Boosting Survival Analysis (GBSA). Each model was adapted for right‐censored survival data and has demonstrated high robustness and accuracy [17]. Model hyperparameters were optimized using the “Optuna” framework, with the concordance index (c‐index) [18] as the evaluation metric. Model performance was assessed and compared on both training and testing sets. The best‐performing model was selected, and Shapley Additive exPlanations (SHAP) values were computed to interpret feature importance and identify key predictors [19].

### 2.5 Risk Score Development and Validation

Based on SHAP values from the final model, we developed an integer‐based risk scoring system to stratify de novo PD patients into risk groups. The top 20 predictors identified by SHAP analysis were dichotomized using median‐based thresholds. Spearman correlation distinguished risk factors (positively correlated) from protective factors (negatively correlated), enhancing clinical applicability. Points were assigned proportionally to each predictor's SHAP importance. The contribution of each feature was calculated as follows:

$$\text{Score}=\left(\frac{\text{Importance of feature in SHAP}}{\text{Total importance of SHAP}}\right)\times 20$$

The total risk score for each participant was computed by summing all assigned feature points.

Participants were classified into low, intermediate, and high‐risk groups based on tertile–based score stratification (33rd and 67th percentiles). The efficacy of this risk stratification was evaluated using Kaplan–Meier curves, with the multivariate log‐rank test applied to contrast the progression of dPD across these groups. Predictive accuracy was assessed by calculating time‐dependent receiver operating characteristic (time‐ROC) curves and corresponding area under the curve (AUC) values. All statistical analyses were performed with Python (version 3.10.16). Two‐tailed statistical tests with a significance threshold of α = 0.05.

## 3 Results

### 3.1 Participants and Baseline Characteristics

A schematic of the inclusion process and cohort classification is shown in Figure 1. A total of 1819 de novo PD participants were initially identified from the PPMI cohort. After applying the inclusion criteria–specifically selecting patients in the “OFF” medication state (excluding *n* = 618) and requiring at least three follow‐up assessments over 1 year (excluding *n* = 41) 579 participants remained. Of these, 18 (3.1%) were excluded due to missing data in predictor variables. After excluding indeterminate cases (*n* = 65), the final analytical cohort consisted of 496 participants. We compared the baseline characteristics of the excluded group and the included group and reported the specific proportion of missing data (Tables S2 and S3). We also conducted a sensitivity analysis using the multiple interpolation method, which further verified the robustness and reliability of the model results (Table S4). The median follow–up was 7 years (IQR: 5–11), with a median of 9 assessment visits (IQR: 7–12). The median age was 62 years (IQR: 55–69), and 304 (61.3%) were male. Among these, 402 (81.0%) were non‐progressors and 94 (19.0%) were progressors. Selected baseline characteristics are summarized in Table 1; others are detailed in Table S5. Significant differences were observed between progressors and non‐progressors across several domains: sleep quality (RBDSQ), cognitive function (BJLOT, SDMT), baseline depression (GDS–15), autonomic function (gastrointestinal and thermoregulatory SCOPA‐AUT scores), and MDS‐UPDRS I (depressed mood, pain and other sensations) (all *p* < 0.001). Progressors exhibited more severe impairments across these measures compared to non‐progressors. The training (*n* = 347, 70%) and testing (*n* = 149, 30%) sets showed comparable baseline characteristics (Table S6).

**TABLE 1:** Baseline characteristics of the non‐progressors and progressors groups.

Variable	All(*n* = 496)	Non‐progressors (*n* = 402)	Progressors (*n* = 9)	*p* Value
Baseline GDS‐15 scores	3.00 (3.00, 4.00)	3.00 (3.00, 3.00)	4.00 (3.00, 4.75)	< 0.001*
Age at Enrollment	62 (55, 69)	62 (55, 69)	64 (55, 69)	0.810
Duration	184 (91,638)	184.00 (91.00, 638.75)	228.00 (92.00, 630.25)	0.565
Follow‐up time	7.00 (5.00, 11.00)	7.00 (5.00, 11.00)	8.00 (6.00, 11.00)	0.129
Time to event	7 (4, 11)	7 (5, 11)	4 (2, 6)	< 0.001*
Sex				0.907
Female	192 (38.7%)	155 (38.6%)	37 (39.4%)	
Male	304 (61.3%)	24 (61.4%)	57 (60.6%)	
Hoehn & Yahr stage				0.703
0	1 (0.2%)	1 (0.2%)	0	
1	196 (39.5%)	162 (40.3%)	34 (36.2%)	
2	287 (57.9%)	229 (57.0%)	58 (61.7%)	
3	12 (2.4%)	10 (2.5%)	2 (2.1%)	
Depression medication				0.198
0	415 (83.7%)	341 (84.8%)	74 (78.7%)	
1	81 (16.3%)	61 (15.2%)	20 (21.3%)	
ESS score	5.00 (3.00, 8.00)	5.00 (3.00, 8.00)	6.00 (4.00, 9.00)	0.030*
RBDSQ score	4.00 (2.00, 6.00)	4.00 (2.00, 6.00)	5.00 (3.25, 7.00)	< 0.001*
SCOPA‐AUT				
Gastrointestinal score	2.00 (0.00, 4.00)	2.00 (0.00, 3.00)	3.00 (1.00, 5.75)	< 0.001*
Urinary score	4.00 (2.00, 6.00)	4.00 (2.00, 5.00)	4.00 (3.00, 7.00)	0.005*
Cardiovascular score	0.00 (0.00, 1.00)	0.00 (0.00, 1.00)	0.50 (0.00, 1.00)	0.008*
Thermoregulatory score	1.00 (0.00,2.00)	1.00 (0.00,2.00)	2.00 (0.25,3.00)	< 0.001*
BJLOT total raw score	13.00 (11.00, 14.00)	13.00 (12.00, 14.00)	12.00 (10.00, 14.00)	< 0.001*
QUIP‐CS score	4.00 (0.00, 4.00)	4.00 (0.00, 4.00)	4.00 (0.25, 4.00)	0.670
SDMT score	42.00 (33.00, 48.00)	42.00 (35.00, 48.00)	38.00 (29.25, 45.00)	0.003*
MDS‐UPDRS I				
Depressed moods	0.00 (0.00, 1.00)	0.00 (0.00, 0.00)	0.00 (0.00, 1.00)	< 0.001*
Sleep problems night	1.00 (0.00, 2.00)	1.00 (0.00, 2.00)	1.00 (0.00, 2.00)	0.047*
Pain and other sensations	1.00 (0.00, 1.00)	0.50 (0.00, 1.00)	1.00 (0.00, 2.00)	< 0.001*

*Note:* Values are *n* (%), mean ± SD, or median (Q1–Q3), unless otherwise indicated.

Abbreviations: BJLOT, benton judgment of line orientation test; ESS, epworth sleepiness scale; LNS, letter‐number sequencing test; MDS‐UPDRS, movement disorder society revision of the unified parkinson disease rating scale; SFT, modified semantic fluency test; RBDSQ, rapid eye movement sleep behavior disorder screening questionnaire; SCOPA‐AUT, scales for outcomes in Parkinson's disease‐autonomic questionnaire; SDMT, symbol digit modalities test.

### 3.2 Performance of Machine Learning Models

The predictive performance of four machine learning models‐XGBoost, RSF, SSVM, and GBSA‐was evaluated using all 72 candidate predictors. All model hyperparameters were optimized using 5‐fold cross‐validation and the “Optuna” framework. The hyperparameter space and final hyperparameter of the four models were detailed in Tables S7 and S8. Initially, RSF and GBSA showed robust accuracy (C‐index = 0.747, 0.731), while XGBoost (C‐index = 0.677) and SSVM (C‐index = 0.636) performed suboptimally in the test set, likely due to high dimensionality relative to sample size. After univariate screening (*p* < 0.1) reduced the predictor set to 22 variables (detailed in Supporting Information, Variables selected of XGBoost and SSVM), both XGBoost (C‐index = 0.637) and SSVM (C‐index = 0.695) did not show improvement in the test set, demonstrating that these two models are not very suitable for this research.

As summarized in Table 2, models using only the top 20 predictors performed comparably to those using the full features, indicating potential for simplification without losing accuracy. Among all models, RSF consistently demonstrated the best performance, supporting its use for subsequent clinical translation. We conducted a calibration assessment and clinical decision analysis on the RSF model, and the results showed that it had good predictive performance and significant clinical net benefit. The detailed results can be found in the Supporting Information (Figure S1 and Figure S2 and Table S9). To assess the impact of the competitive event, we also conducted a sensitivity analysis, and the results showed that it had a negligible effect on the stability of the risk stratification in this study (Figure S3 and Table S10).

**TABLE 2:** Comparing the predictive performance of different models.

C‐index	XGBoost	RSF	SSVM	GBSA
Models trained with all variables				
Train set	0.928 (95% CI: 0.899–0.953)	0.890 (95% CI: 0.889–0.891)	0.813 (95% CI: 0.770–0.854)	0.837 (95% CI: 0.792–0.876)
Test set	0.637 (95% CI: 0.528–0.740)	0.747 (95% CI: 0.744–0.749)	0.695 (95% CI: 0.614–0.783)	0.731 (95% CI: 0.650–0.814)
Models trained with top 20 variables				
Train set	0.842 (95% CI: 0.797–0.879)	0.873 (95% CI: 0.872–0.874)	0.813 (95% CI: 0.770–0.854)	0.826 (95% CI: 0.774–0.873)
Test set	0.629 (95% CI: 0.498–0.756)	0.745 (95% CI: 0.742–0.748)	0.695 (95% CI: 0.616–0.784)	0.791 (95% CI: 0.710–0.865)

Abbreviations: C‐index, concordance index; GBSA, gradient boosting survival analysis; SF, random survival forest; SSVM, survival support vector machine; XGBoost, extreme gradient boosting.

### 3.3 Feature Contribution and Clinically Relevant Thresholds Identified by SHAP

**FIGURE 2:**
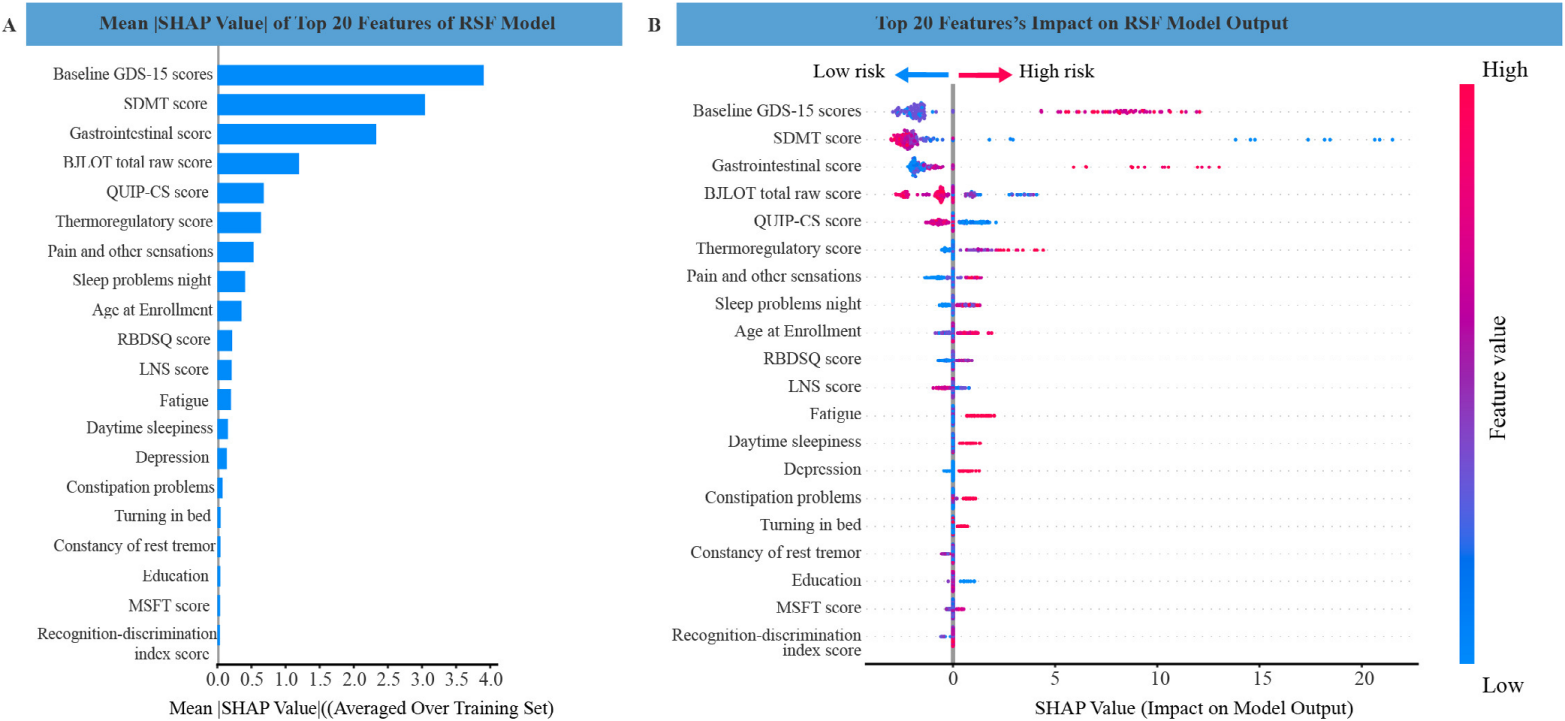
The summary plot of SHAP: (A) SHapley Additive exPlanations (SHAP) summary plot shows the top 20 features contributing to the initial RSF model's prediction of dPD progression. Features are positioned along the y axis based on importance. (B) The location of each feature on the y‐axis ranks its importance according to the model prediction in descending order. For each feature, one dot represents a single patient and the dot's color is an indicator of that feature value, where blue represents the lower value and red represents the higher value. A dot's position along the x‐axis (i.e., the actual SHAP value) illustrates the impact that the feature had on the model's output (positively or negatively) for that specific patient. Mathematically, this corresponds to the (logarithm of the) likelihood of dPD progression relative across patients (i.e., a patient with a higher SHAP value has a higher likelihood of dPD progression relative to a patient with a lower SHAP value).

To quantify individual predictors' importance and identify clinically actionable thresholds, SHAP analysis was conducted using the RSF model, given its superior predictive performance and inherent compatibility with SHAP for interpreting feature contributions. Figure 2A showed the mean SHAP value of the top 20 predictors in the RSF model. Figure 2B presented how these predictors affected the model's output. These key predictive features predominantly encompassed non–motor symptoms, notably depressive symptom severity (baseline GDS‐15 scores, SHAP = 3.9040), cognitive impairment (SDMT score [SHAP = 3.0416], BJLOT total raw score [SHAP = 1.1980]), autonomic dysfunction (gastrointestinal [SHAP = 2.3288] and thermoregulatory subscores [SHAP = 0.6400]), impulse control disorder (QUIP‐CS score, SHAP = 0.6785), MDS‐UPDRS I (pain and other sensations [SHAP = 0.5313], sleep problems night [SHAP = 0.4068]), and age (SHAP = 0.3530).

**FIGURE 3:**
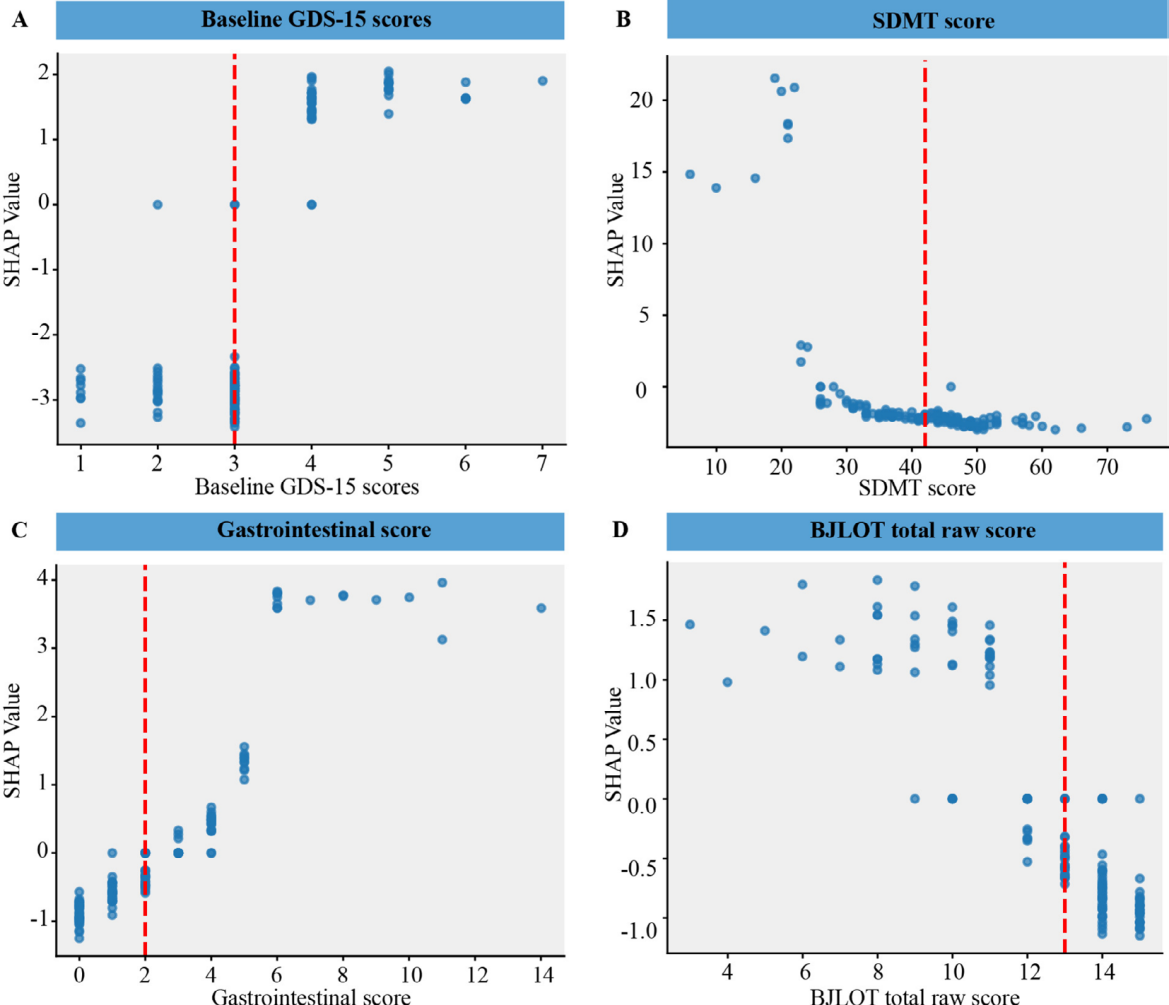
SHAP feature dependence plots: The SHAP dependence plot shows how a single feature affects the output of the prediction model, as seen with baseline GDS‐15 scores (A) SDMT score (B) gastrointestinal score (C) and BJLOT total raw score (D) SHAP values for specific features exceed zero, representing an increased likelihood of dPD progression. The x‐axis represents the feature value, and the y‐axis represents the feature attribution to the predicted result. Each data point corresponds to 1 prediction from a particular patient. The red dotted lines represent the optimal threshold for each feature determined based on the median of the total data. Abbreviations as in Table 1. SDMT, Symbol Digit Modalities Test, BJLOT, Benton Judgment of Line Orientation Test.

SHAP dependence plots delineated feature‐specific associations with depressive progression risk. Specifically, a positive linear relationship was observed between baseline GDS–15 scores and its SHAP values, indicating higher progression risk with more severe depression (Figure 3A). Furthermore, inflection points were identified for autonomic dysfunction scores, revealing sharply increased progression risk beyond clinically relevant thresholds: gastrointestinal score > 2 (Figure 3C). Conversely, a negative linear correlation was noted between feature values and corresponding SHAP values, both in SDMT score and BJLOT total raw score, indicating lower depressive progression rates with better cognitive performance (Figure 3B, Figure 3D). Median‐based cut‐off thresholds derived from the entire dataset were defined as: baseline GDS‐15 scores (> 3), SDMT score (< 42), gastrointestinal score (> 2), and BJLOT total raw score (< 13).

### 3.4 Risk Score Derivation and Validation

**FIGURE 4:**
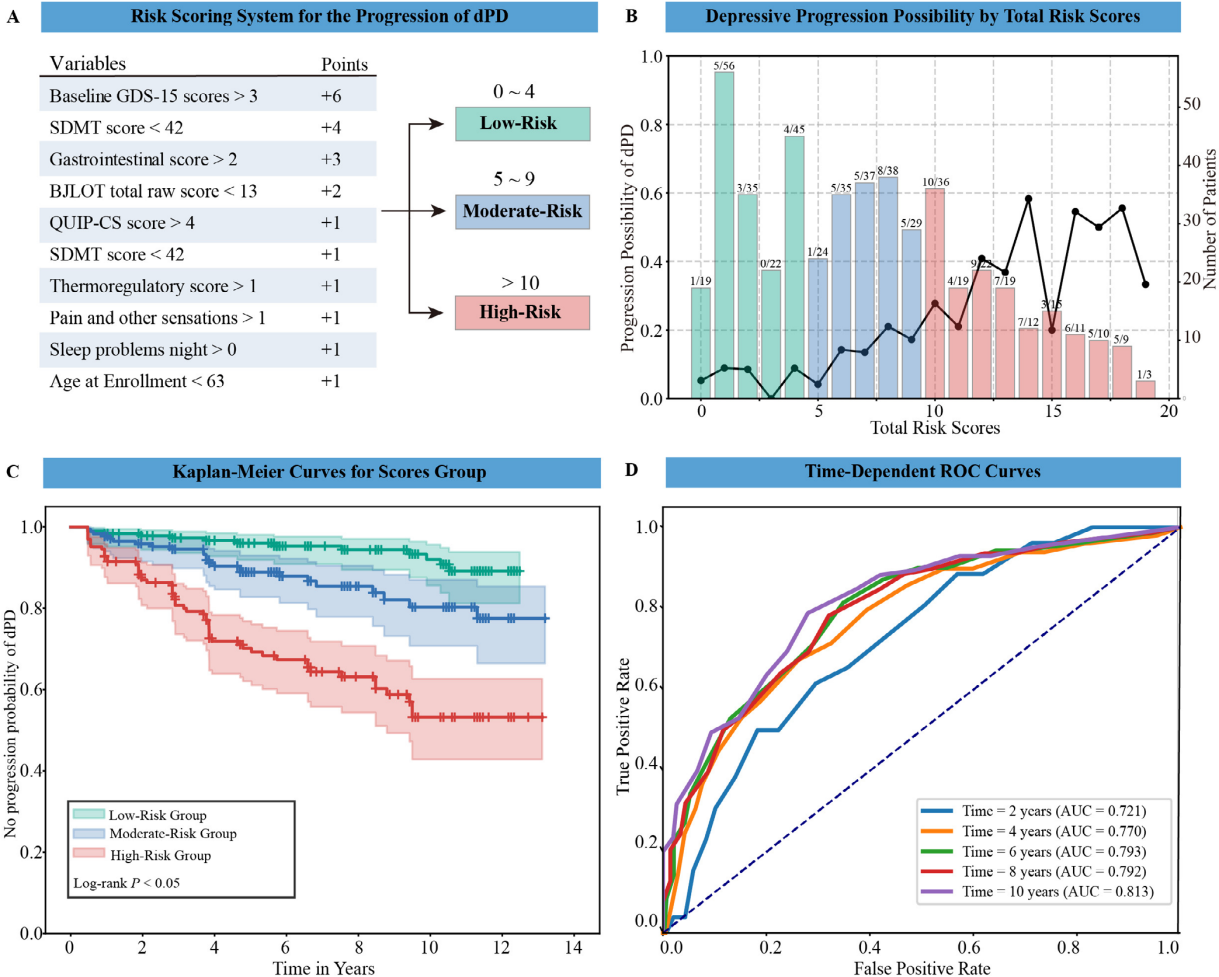
The risk model and model evaluation: (A) Risk scoring system for the progression of dPD. (B) The graph showing the progression rate of dPD in PD patients based on the risk score. (C) The Kaplan–Meier curve plotted based on the predicted risk score. (D) Time ROC curves comparing the performance of the risk model at 2 year, 4 year, 6 year, 8 year, and 10 year follow‐up. dPD, Depressive disturbance in Parkinson's disease.

Based on SHAP values derived from the RSF model, a clinically applicable integer–based risk scoring system was developed, integrating the top 20 predictors. Integer scores were assigned proportionally to each predictor, identifying risk factors (scores assigned if thresholds exceeded: baseline GDS‐15 > 3 [6 points], SDMT < 42 [4 points], gastrointestinal > 1 [3 points], BJLOT < 13 [2 points], QUIP‐CS > 4 [1 point], Thermoregulatory > 1 [1 point], pain and other sensations > 1 [1 point], sleep problems night > 1 [1 point], age < 63 [1 point]) (Figure 4A). The final individual risk score was calculated as the sum of all assigned points.

Using tertile‐based thresholds (33rd and 67th percentiles), patients were stratified into low–risk (score ≤ 4, *n* = 177, progression rate: 7.3%), moderate‐risk (score 5–9, *n* = 163, progression rate: 14.7%), and high‐risk (score ≥ 10, *n* = 156, progression rate: 36.5%) groups. The distribution of depressive progression rates across total risk scores is visualized in Figure 4B, where risk increased monotonically with score. Kaplan–Meier analysis confirmed statistically significant differences in progression‐free probability among these risk strata (log–rank *p* < 0.05, Figure 4C). Time–dependent ROC analysis further confirmed the robust predictive accuracy of this risk scoring system, with AUC values consistently high across multiple time points: 0.721 (2 year), 0.770 (4 year), 0.794 (6 year), 0.792 (8 year), and 0.812 (10 year) (Figure 4D).

## 4 Discussion

We developed and validated an explainable survival model to predict long‐term dPD progression and identify high‐risk patients. Using PPMI data, our risk scoring system accurately predicted depressive progression in de novo PD patients. SHAP analysis revealed that baseline GDS‐15, autonomic function, sleep quality, and cognitive performance were the main predictors. To our knowledge, this is the first study to apply machine‐learning survival analysis and SHAP interpretation to predict long‐term dPD progression and develop an internally validated clinical risk score system.

### 4.1 The Definition of Outcome Indicator

Analyzing longitudinal follow‐up data in PPMI, we defined dPD as a sustained worsening in GDS‐15 category lasting ≥ 12 months without subsequent improvement, to minimize false‐positive diagnoses. Prior investigations predominantly employed dichotomous classification modeling strategies, merely predicting depression occurrence but failing to capture dynamic depressive symptoms changes [7, 8, 9, 10, 11, 12, 13, 14, 15, 16, 17, 18, 19, 20]. In our study, individual risk trajectories across disease progression could be delineated by utilizing RSF survival model, supporting clinical management of dPD. Studies have revealed that individuals with depression are associated with greater emotional variability compared to healthy controls, particularly increased instability in negative emotions [21, 22]. These findings suggested the dynamic nature of depressive symptoms. Therefore, longitudinal analysis across multiple time points can more effectively identify trends in the progression of dPD, thereby facilitating early intervention.

### 4.2 Model Performance

The RSF model performs well in processing high–dimensional data and exhibited intrinsic robustness against overfitting, making it widely used in the medical domain [23, 24]. Previous linear models could only incorporate a limited number of variables, so they usually only included the total score of the rating scale rather than the scores of individual symptom sub–items, such as SCOPA‐AUT, MDS‐UPDRS III, etc. By utilizing the RSF model, we could be considered better in the intricate and heterogeneous nature of the progression of dPD through incorporating more variables.

We identified the most influential features and established clinical thresholds for the risk scoring system with SHAP analysis. Key features included baseline GDS‐15, cognitive function (BJLOT, SDMT), autonomic dysfunction (gastrointestinal and thermoregulatory scores), impulse control disorder (QUIP‐CS score), MDS–UPDRS I (pain and other sensations and sleep problems night), and age. Non–motor symptoms were the primary predictors of dPD progression, while motor symptoms showed negligible contribution and were absent among the top 20 predictors. These findings suggest that dPD progression is more associated with limbic system impairment and neurotransmitter imbalance than with nigrostriatal dysfunction. Early detection and management of non‐motor symptoms may therefore help delay or prevent dPD progression.

### 4.3 Key Predictive Features

Patients with higher baseline GDS‐15 scores (SHAP = 3.8967) were more likely to develop dPD, in accordance with previous studies on dPD [7, 8, 9, 10, 11, 12, 13, 14, 15, 16, 17, 18, 19, 20, 21, 22, 23, 24, 25]. This progression is linked to the “negative bias” within the anterior cingulate–amygdala–hippocampal circuit [26]. Repeated negative emotional processing lowers the amygdala's threshold for threat responses and reduces anterior cingulate inhibition, promoting consolidation of negative memories and formation of a depressive circuit. Prior to the onset of motor symptoms, PD patients have already exhibited neurodegeneration in key neuromodulatory regions, including the raphe nuclei (5–HT), locus coeruleus (NE), and ventral tegmental area (DA). These deficits lower the activation threshold of emotional reinforcement circuits, allowing milder stimuli to trigger negative emotional responses. Consequently, what previously required repeated stressful events to trigger may now be elicited by milder stimuli. Prior studies have indicated that the baseline GDS‐15 score holds predictive value for the progression of dPD. In this study, we quantified its contribution using a risk–scoring system developed with the machine learning and SHAP methodology.

This study assessed various cognitive domains, incorporating global cognition, memory, attention, and language, using several scales. Ultimately SDMT score (attention) and BJLOT total raw score (visual spatial function) were included in the final model. Previous studies have shown that depressive symptoms impair cognitive functions such as working memory, verbal learning, and delayed recall, via disruption of the prefrontal–limbic axis and striatal dopamine depletion [27]. SDMT is associated with the locus coeruleus‐norepinephrine pathway, which may suppress depression‐related behaviors via limbic modulation [27]. The BJLOT task involves a right–hemispheric fronto‐parieto‐occipital network. In an SVM model classifying dPD and ndPD patients, fractional anisotropy in the right superior longitudinal fasciculus III (SLF–III) alone contributed 18% to classification accuracy, highlighting its potential in predicting dPD progression [28].

Gastrointestinal score (SHAP = 2.3349), thermoregulatory score (SHAP = 0.6527) were all utilized to evaluate autonomic nervous function, demonstrating great significance to the final model. Evidence suggests overlapping pathophysiology between dPD and autonomic dysfunction. Key regions such as the hypothalamus, anterior cingulate cortex, medial prefrontal cortex, and insula are involved in both the central autonomic network (CAN), which regulates autonomic activity, and the limbic system, which modulates emotion [29]. Gastrointestinal dysfunction correlates with impaired acetylcholine and 5‐HT signaling in the vagus and enteric nervous system [30], and thermoregulatory dysfunction is linked to acetylcholine depletion in the insular cortex [31]. Our findings suggest that dPD progression is more closely linked to cholinergic and serotonergic systems than to noradrenergic pathways. Furthermore, we propose that autonomic symptoms may not merely be secondary to depression, but represent co‐occurring clinical phenotypes stemming from shared damage within the CAN.

We found that impulse control disorder (QUIP‐CS score, SHAP = 0.6674) can serve as a predictive indicator for dPD progression, and the underlying mechanism involves the dysregulation of the interaction between the dopamine and serotonin systems. Dopaminergically, ventral striatal denervation in PD leads to limbic CSTC circuit imbalance, with reduced activity in depression and increased activity in impulse control disorder post‐dopamine replacement therapy [32]. Serotonergically, impulse control disorder patients show preserved SERT binding in the posterior putamen and upregulated cortical 5–HT2A receptors, impairing behavioral inhibition and emotional regulation [33].

Previous study has found a correlation between the overall score of MDS–UPDRS Part I and dPD progression [34]. In our study, we identified two specific sub‐items, pain and other sensations (SHAP = 0.5352) and sleep problems night (SHAP = 0.4176), as key predictors for dPD. This suggests that beyond the global non‐motor symptom burden, dynamic changes in these specific domains are critical for assessing dPD progression. Prior research identified microstructural deterioration in hypothalamic subunits in patients with REM sleep behavior disorder [35], and linked higher RBDSQ scores to elevated α–synuclein oligomer levels in CSF, affecting emotional regulation via the limbic system [36]. We propose that sleep problems night and depression may share underlying limbic circuit mechanisms, which could facilitate the prediction of dPD progression.

In summary, dPD progression primarily involves limbic system impairment, along with acetylcholine and serotonin depletion and disruption of the locus coeruleus–norepinephrine pathway. Clinically accessible features such as baseline GDS–15, thermoregulatory dysfunction, and gastrointestinal symptoms predict dPD progression, as they reflect shared abnormalities in neural circuits and neurotransmitter systems.

### 4.4 Potential Clinical and Research Significance

We developed a clinically applicable risk scoring system based on SHAP values to predict dPD progression. To our knowledge, this is the first model that provides individualized risk stratification, demonstrating strong predictive performance and time–dependent ROC accuracy. The system uses routinely collected clinical variables without requiring additional tests, enhancing its generalizability and practicality.

Clinically, this tool enables early risk stratification in PD, facilitating the development of personalized monitoring and intervention strategies. For high–risk patients, we propose a proactive, multi‐tiered management framework. We recommend enhanced screening via instruments like the HAMD and prioritizing non‐pharmacological interventions, such as Cognitive Behavioral Therapy (CBT), to avoid exacerbating motor or autonomic symptoms [37]. Regarding pharmacotherapy, while SSRIs are preferred, caution is warranted with Tricyclic Antidepressants (TCAs) in patients with baseline autonomic dysfunction‐a key predictor in our model‐to prevent worsening gastrointestinal or thermoregulatory issues. Conversely, low‐risk patients may avoid unnecessary visits, thereby optimizing healthcare resource allocation. Ultimately, risk communication can increase patient awareness and treatment adherence, fostering more effective long‐term management of dPD.

From a research standpoint, the system offers a rational approach to participant enrichment in clinical trials. Selecting high‐risk individuals may shorten trial duration, reduce sample sizes, and improve statistical power. Furthermore, SHAP‐based feature contributions provide mechanistic insights that help bridge clinical observation with basic research, supporting advances in precision medicine for dPD.

## 5 Limitations

There are several limitations to our study. Although the final model showed strong performance in internal validation and effectively stratified patients by risk, its generalizability requires confirmation through multicenter external validation. Another limitation is the relatively low ratio of events to variables (EPV = 1.3), given the 94 progression events and 72 initial predictors. This high dimensionality relative to the event count poses a potential risk of overfitting. However, our sensitivity analysis demonstrated that a simplified model using the top 20 predictors yielded comparable performance, suggesting the core predictive findings are stable. Furthermore, while the model identifies high‐risk individuals and key prognostic factors, it does not provide specific clinical strategies to intervene against dPD progression. Future prospective studies should investigate whether early targeted interventions based on this model can improve outcomes. Although the model incorporated demographic, motor, and non–motor variables, it did not include medication history of dopamine, biomarkers, or neuroimaging data‐all available in PPMI and potentially relevant to dPD progression. Specifically, dopamine agonists‐common in PD management‐are known to exert dual effects: they possess potential antidepressant properties via the mesolimbic pathway but are also closely linked to the development of impulse control disorders [37]. The absence of these pharmacological variables might confound the model's ability to purely reflect the natural progression of dPD. Additionally, data on genetic variants (e.g., SLC6A15, TPH2, LRRK2) were unavailable in PPMI [38]. Including these variables in future iterations may enhance predictive accuracy.

## 6 Conclusion

We developed and internally validated a machine learning‐based survival model for predicting long‐term progression of dPD. The model, supported by SHAP analysis, identified key clinical predictors and enabled effective patient stratification. This risk score has the potential to guide early intervention and personalized management, although external validation is required before clinical implementation.

## Author Contributions

Conception and design: Hutao Xie, Jiansong Huang, Defu Liu, Yin Jiang, Jianguo Zhang. Acquisition of data: Defu Liu, Chong Qi, Jiansong Huang, Hutao Xie, Yutong Zhuang, Quan Zhang, Tianqi Hu, Xin Zhang, Guofan Qin, Yue Lu. Analysis and interpretation of data: Defu Liu, Jiansong Huang, Hutao Xie, Yutong Zhuang, Sizhe Li, Xuemin Zhao, Mingyu Yang. First draft of manuscript: Defu Liu, Chong Qi, Jiansong Huang. Revision of manuscript: Hutao Xie, Jiansong Huang, Chong Qi, Defu Liu, Yin Jiang, Jianguo Zhang. All authors contributed substantially to this research.

## Funding

This work was supported by the National Natural Science Foundation of China (Nos. 82401713, 82371256, 82301655, 81830033) and the China Postdoctoral Science Foundation (No. GZC20231742).

## Disclosure

Code Sharing: The code for model establishment and analysis has been submitted in the form of an attachment.

## Ethics Statement

PPMI is an ongoing, multicenter, prospective observational study, registered at ClinicalTrials.gov (Identifier: NCT01141023). All participants provided written informed consent, and ethical approval was obtained from institutional review boards of all participating centers. Detailed study protocols and data access information are available at www.ppmi‐info.org.

## Consent

The authors have nothing to report.

## Conflicts of Interest

The authors declare no conflicts of interest.

## Supporting information


**Figure S1:** Test set calibration analysis (RSF model).
**Figure S2:** Test set DCA analysis (RSF model).
**Figure S3:** Sensitivity analysis: impact of competing risks.
**Table S1:** Candidate predictors included in the study.
**Table S2:** Comparison of baseline features between the included set and the excluded set.
**Table S3:** Missing data distribution.
**Table S4:** Sensitivity analysis of model performance: comparison between complete‐case analysis and multiple imputation.
**Table S5:** Baseline characteristics of the non‐progressors and progressors groups.
**Table S6:** Comparison of baseline features between the training set and the test set.
**Table S7:** Hyperparameter space of the models.
**Table S8:** Hyperparameter of the models.
**Table S9:** Sensitivity analysis of model performance: comparison between complete‐case analysis and multiple imputation.
**Table S10:** Comparison of Aalen‐johansen estimation of cumulative incidence rate of Events under competing risks analysis with Kaplan–Meier estimation.

## Data Availability

The data that support the findings of this study are openly available in PPMI at https://www.ppmi‐info.org/access%E2%80%90data%E2%80%90specimens/download%E2%80%90data, reference number NCT04477785.

## References

[1] J. S. Reijnders , U. Ehrt , W. E. Weber , et al., “A Systematic Review of Prevalence Studies of Depression in Parkinson's Disease,” Movement Disorders 23, no. 2 (2008): 183–189, 10.1002/mds.21803. 17987654

[2] N. A. Pachana , S. J. Egan , K. Laidlaw , et al., “Clinical Issues in the Treatment of Anxiety and Depression in Older Adults With Parkinson's Disease,” Movement Disorders 28, no. 14 (2013): 1930–1934, 10.1002/mds.25689. 24123116

[3] S. Wang , S. Mao , D. Xiang , and C. Fang , “Association Between Depression and the Subsequent Risk of Parkinson's Disease: A Meta‐Analysis,” Progress in Neuro‐Psychopharmacology & Biological Psychiatry 86 (2018): 186–192, 10.1016/j.pnpbp.2018.05.025. 29859854

[4] C. G. Goetz , “New Developments in Depression, Anxiety, Compulsiveness, and Hallucinations in Parkinson's Disease,” Movement Disorders 25, no. 1 (2010): S104–S109, 10.1002/mds.22636. 20187250

[5] B. Ravina , R. Camicioli , P. G. Como , et al., “The Impact of Depressive Symptoms in Early Parkinson Disease,” Neurology 69, no. 4 (2007): 342–347, 10.1212/01.wnl.0000268695.63392.10. 17581943 PMC2031220

[6] R. Ou , Q. Wei , Y. Hou , et al., “Vascular Risk Factors and Depression in Parkinson's Disease,” European Journal of Neurology 25, no. 4 (2018): 637–643, 10.1111/ene.13551. 29271534

[7] S. C. Gu , J. Zhou , C. X. Yuan , and Q. Ye , “Personalized Prediction of Depression in Patients With Newly Diagnosed Parkinson's Disease: A Prospective Cohort Study,” Journal of Affective Disorders 268 (2020): 118–126, 10.1016/j.jad.2020.02.046. 32158001

[8] A. Yesilyaprak , A. K. Kumar , A. Agrawal , et al., “Predicting Long‐Term Clinical Outcomes of Patients With Recurrent Pericarditis,” Journal of the American College of Cardiology 84, no. 13 (2024): 1193–1204, 10.1016/j.jacc.2024.05.072. 39217549

[9] The Parkinson Progression Marker Initiative (PPMI),” Progress in Neurobiology 95, no. 4 (2011): 629–635, 10.1016/j.pneurobio.2011.09.005. 21930184 PMC9014725

[10] D. Weintraub , K. Saboe , and M. B. Stern , “Effect of Age on Geriatric Depression Scale Performance in Parkinson's Disease,” Movement Disorders 22, no. 9 (2007): 1331–1335, 10.1002/mds.21369. 17546674 PMC2000798

[11] National Collaborating Centre for Mental Health , Depression in Adults With a Chronic Physical Health Problem: Treatment and Management (British Psychological Society and the Royal College of Psychiatrists, 2010). 22259826

[12] National Institute for Health and Care Excellence: Guidelines , Depression in Adults: Treatment and Management (National Institute for Health and Care Excellence (NICE), 2022). 35977056

[13] C. Shin , M. H. Park , S. H. Lee , et al., “Usefulness of the 15‐Item Geriatric Depression Scale (GDS‐15) for Classifying Minor and Major Depressive Disorders Among Community‐Dwelling Elders,” Journal of Affective Disorders 259 (2019): 370–375, 10.1016/j.jad.2019.08.053. 31470180

[14] D. Weintraub , K. A. Oehlberg , I. R. Katz , and M. B. Stern , “Test Characteristics of the 15‐Item Geriatric Depression Scale and Hamilton Depression Rating Scale in Parkinson Disease,” American Journal of Geriatric Psychiatry 14, no. 2 (2006): 169–175, 10.1097/01.JGP.0000192488.66049.4b. PMC1571046 16473982

[15] A. F. Leentjens , A. J. Moonen , K. Dujardin , et al., “Modeling Depression in Parkinson Disease: Disease‐Specific and Nonspecific Risk Factors,” Neurology 81, no. 12 (2013): 1036–1043, 10.1212/WNL.0b013e3182a4a503. 23946309 PMC3795592

[16] N. N. Dissanayaka , J. D. O'sullivan , P. A. Silburn , and G. D. Mellick , “Assessment Methods and Factors Associated With Depression in Parkinson's Disease,” Journal of the Neurological Sciences 310, no. 1–2 (2011): 208–210, 10.1016/j.jns.2011.06.031. 21764079

[17] P. Wang , Y. Li , and C. K. Reddy , “Machine Learning for Survival Analysis: A Survey,” ACM Computing Surveys 51, no. 6 (2019): 110:1–110:36, 10.1145/3214306.

[18] T. Akiba , S. Sano , T. Yanase , et al., “Optuna: A Next‐generation Hyperparameter Optimization Framework,” (2019), 10.1145/3292500.3330701.

[19] L. S. Shapley , “A Value for n‐Person Games,” in Contributions to the Theory of Games, vol. 2, ed. K. Harold William and T. Albert William (Princeton University Press, 1953), 307–318.

[20] H. Zhang , Y. Zhang , and G. Li , “Machine Learning Study on Predicting Depressive Symptoms and Genetic Correlations in Parkinson's Disease,” Frontiers in Aging Neuroscience 17 (2025): 1584005, 10.3389/fnagi.2025.1584005. 40271183 PMC12014618

[21] O. V. Ebrahimi , D. Borsboom , R. H. A. Hoekstra , et al., “Towards Precision in the Diagnostic Profiling of Patients: Leveraging Symptom Dynamics as a Clinical Characterisation Dimension in the Assessment of Major Depressive Disorder,” British Journal of Psychiatry 224, no. 5 (2024): 157–163, 10.1192/bjp.2024.19. PMC11039556 38584324

[22] T. Hu , H. Xie , Y. Diao , et al., “Effects of Subthalamic Nucleus Deep Brain Stimulation on Depression in Patients With Parkinson's Disease,” Journal of Clinical Medicine 11, no. 19 (2022): 5844, 10.3390/jcm11195844. 36233710 PMC9572818

[23] E. Audureau , F. Carrat , R. Layese , et al., “Personalized Surveillance for Hepatocellular Carcinoma in Cirrhosis ‐ Using Machine Learning Adapted to HCV Status,” Journal of Hepatology 73, no. 6 (2020): 1434–1445, 10.1016/j.jhep.2020.05.052. 32615276

[24] B. Jamet , L. Morvan , C. Nanni , et al., “Random Survival Forest to Predict Transplant‐Eligible Newly Diagnosed Multiple Myeloma Outcome Including FDG‐PET Radiomics: A Combined Analysis of Two Independent Prospective European Trials,” European Journal of Nuclear Medicine and Molecular Imaging 48, no. 4 (2021): 1005–1015, 10.1007/s00259-020-05049-6. 33006656

[25] Z. Goodarzi , K. J. Mrklas , D. J. Roberts , N. Jette , T. Pringsheim , and J. Holroyd‐Leduc , “Detecting Depression in Parkinson Disease: A Systematic Review and Meta‐Analysis,” Neurology 87, no. 4 (2016): 426–437, 10.1212/wnl.0000000000002898. 27358339 PMC4977107

[26] M. Klug , V. Enneking , T. Borgers , et al., “Persistence of Amygdala Hyperactivity to Subliminal Negative Emotion Processing in the Long‐Term Course of Depression,” Molecular Psychiatry 29, no. 5 (2024): 1501–1509, 10.1038/s41380-024-02429-4. 38278993 PMC11189807

[27] J. D. Jones , N. E. Kurniadi , T. P. Kuhn , S. M. Szymkowicz , J. Bunch , and E. Rahmani , “Depressive Symptoms Precede Cognitive Impairment in de Novo Parkinson's Disease Patients: Analysis of the PPMI Cohort,” Neuropsychology 33, no. 8 (2019): 1111–1120, 10.1037/neu0000583. 31343240 PMC6823115

[28] Y. Yang , Y. Yang , A. Pan , et al., “Identifying Depression in Parkinson's Disease by Using Combined Diffusion Tensor Imaging and Support Vector Machine,” Frontiers in Neurology 13 (2022): 878691, 10.3389/fneur.2022.878691. 35795798 PMC9251067

[29] E. Dayan , M. Sklerov , and N. Browner , “Disrupted Hypothalamic Functional Connectivity in Patients With PD and Autonomic Dysfunction,” Neurology 90, no. 23 (2018): e2051–e2058, 10.1212/wnl.0000000000005641. 29728527

[30] D. Q. Zhao , H. Xue , and H. J. Sun , “Nervous Mechanisms of Restraint Water‐Immersion Stress‐Induced Gastric Mucosal Lesion,” World Journal of Gastroenterology 26, no. 20 (2020): 2533–2549, 10.3748/wjg.v26.i20.2533. 32523309 PMC7265141

[31] S. S. Nair , M. M. Govindankutty , M. Balakrishnan , K. Prasad , T. N. Sathyaprabha , and K. Udupa , “Investigation of Autonomic Dysfunction in Alzheimer's Disease‐A Computational Model‐Based Approach,” Brain Sciences 13, no. 9 (2023): 1322, 10.3390/brainsci13091322. 37759923 PMC10526304

[32] C. Vriend , T. Pattij , Y. D. VAN DER Werf , et al., “Depression and Impulse Control Disorders in Parkinson's Disease: Two Sides of the Same Coin?,” Neuroscience and Biobehavioral Reviews 38 (2014): 60–71, 10.1016/j.neubiorev.2013.11.001. 24239733

[33] S. Prange , E. Metereau , H. Klinger , et al., “Serotonergic Dysfunction in Patients With Impulse Control Disorders in Parkinson's Disease,” Brain 148, no. 6 (2025): 2108–2121, 10.1093/brain/awaf087. 40042882 PMC12129733

[34] G. W. Duncan , T. K. Khoo , A. J. Yarnall , et al., “Health‐Related Quality of Life in Early Parkinson's Disease: The Impact of Nonmotor Symptoms,” Movement Disorders 29, no. 2 (2014): 195–202, 10.1002/mds.25664. 24123307

[35] C. Zhou , J. You , X. Guan , et al., “Microstructural Alterations of the Hypothalamus in Parkinson's Disease and Probable REM Sleep Behavior Disorder,” Neurobiology of Disease 194 (2024): 106472, 10.1016/j.nbd.2024.106472. 38479482

[36] Y. Hu , S. Y. Yu , L. J. Zuo , et al., “Parkinson Disease With REM Sleep Behavior Disorder: Features, α‐Synuclein, and Inflammation,” Neurology 84, no. 9 (2015): 888–894, 10.1212/wnl.0000000000001308. 25663225

[37] F. Assogna , C. Pellicano , C. Savini , et al., “Drug Choices and Advancements for Managing Depression in Parkinson's Disease,” Current Neuropharmacology 18, no. 4 (2020): 277–287, 10.2174/1570159x17666191016094857. 31622207 PMC7327944

[38] J. Zheng , X. Yang , Q. Zhao , et al., “Association Between Gene Polymorphism and Depression in Parkinson's Disease: A Case–Control Study,” Journal of the Neurological Sciences 375 (2017): 231–234, 10.1016/j.jns.2017.02.001 28320136

