# Supplementary material for: Predicting Long‐Term Depression Progression in Parkinson's Disease: A Machine‐Learning Survival Analysis and Risk Score
Source: CNS Neurosci Ther. 2026 Mar 28;32(4):e70845. doi: 10.1002/cns.70845 (PMC13140941; doi:10.1002/cns.70845)
Supplement: Supplementary file 1 — Figure S1: Test set calibration analysis (RSF model). Figure S2: Test set DCA analysis (RSF model). Figure S3: Sensitivity analysis: impact of competing risks. Table S1: Candidate predictors included in the study. Table S2: Comparison of baseline features between the included set and the excluded set. Table S3: Missing data distribution. Table S4: Sensitivity analysis of model performance: comparison between complete‐case analysis and multiple imputation. Table S5: Baseline characteristics of the non‐progressors and progressors groups. Table S6: Comparison of baseline features between the training set and the test set. Table S7: Hyperparameter space of the models. Table S8: Hyperparameter of the models. Table S9: Sensitivity analysis of model performance: comparison between complete‐case analysis and multiple imputation. Table S10: Comparison of Aalen‐johansen estimation of cumulative incidence rate of Events under competing risks analysis with Kaplan–Meier estimation. [file CNS-32-e70845-s001.docx]

**Supplementary Materials**

**Supplementary Table 1. Candidate Predictors Included in the Study**

| Assessment | Candiate predictors |
| --- | --- |
| Demographic variables | Age at Enrollment |
|  | Education |
|  | Duration |
|  | Sex |
|  | Handedness |
|  | Depression |
|  | Symptom onset side |
|  | Depression medication |
| Depressive symptom severity | Baseline GDS-15 scores |
| The severity of PD | Hoehn & Yahr stage |
| Autonomy and efficiency in daily activities | MSEADL score |
| Sleep assessment | ESS score |
|  | RBDSQ score |
| Autonomic nervous function | SCOPA-AUT |
|  | Gastrointestinal score |
|  | Urinary score |
|  | Cardiovascular score |
|  | Thermoregulatory score |
|  | Pupillomotor score |
|  | Sexual score |
| Impulse control | QUIP-CS score |
| Emotion state | STAI score |
| Cognitive function | HVLT-R |
|  | Total recall score |
|  | Delayed recall score |
|  | Retention score |
|  | Recognition-discrimination index score |
|  | LNS score |
|  | BJLOT total raw score |
|  | MSFT score |
|  | SDMT score |
| Non-motor experiences of daily living(subitem of MDS-UPDRS I) | MDS-UPDRS I |
|  | Congitive impairment |
|  | Hallucinations and psychosis |
|  | Depressed moods |
|  | Anxious mood |
|  | Apathy |
|  | Features dopamine dysregulation syndrome |
|  | Sleep problems night |
|  | Daytime sleepiness |
|  | Pain and other sensations |
|  | Urinary problems |
|  | Constipation problems |
|  | Lightheadedness on standing |
|  | Fatigue |
| Motor experiences of daily living(subitem of MDS-UPDRS II) | MDS-UPDRS II |
|  | Speech |
|  | Saliva drooling |
|  | Chewing and swallowing |
|  | Eating tasks |
|  | Dressing |
|  | Hygiene |
|  | Handwriting |
|  | Doing hobbies and other activities |
|  | Turning in bed |
|  | Tremor |
|  | Getting out of bed car or deep chair |
|  | Walking and balance |
|  | Freezing |
| Motor examination(subitem of MDS-UPDRS III) | MDS-UPDRS III |
|  | Speech |
|  | Facial expression |
|  | Rigidity |
|  | Finger tapping |
|  | Hand movements |
|  | Pronation supination |
|  | Toe tapping |
|  | Leg agility |
|  | Arising from chair |
|  | Gait |
|  | Freezing of gait |
|  | Postural stability |
|  | Posture |
|  | Global spontaneity of movement |
|  | Postural tremor |
|  | Kinetic tremor |
|  | Rest tremor amplitude |
|  | Constancy of rest tremor |

**Schematic of the inclusion process**

A total of 1,819 de novo PD participants were initially identified from the PPMI cohort. After applying the inclusion criteria—specifically selecting patients in the 'OFF' medication state (excluding n=618) and requiring at least three follow-up assessments over one year (excluding n=41)—579 participants remained. Of these, 18 (3.1%) were excluded due to missing data in predictor variables. After excluding indeterminate cases (n=65), the final analytical cohort consisted of 496 participants.

We conducted a systematic comparison of baseline characteristics between the included (n=496) and excluded (n=618) groups. These results are now detailed in Supplementary Table 1. The analysis shows:

Baseline Psychological Stability: There was no significant difference in the primary measure, baseline GDS-15 scores (p=0.241), or in most cognitive and impulse-control metrics, ensuring that the two groups were psychologically comparable at baseline.

Reasoning for Differences: Significant differences in motor severity (MDS-UPDRS III) and disease duration were primarily attributed to our strict inclusion criteria requiring the 'OFF' medication state and long-term longitudinal follow-up. Naturally, patients with longer duration or more advanced symptoms were more likely to be on medication or have incomplete 'OFF' state data, leading to their exclusion.

Robustness despite Attrition: Although the excluded group was slightly older and had shorter follow-up, the consistency in baseline depression levels and non-motor profiles suggests that our model's predictions were not fundamentally skewed by these demographic shifts.

**Supplementary Table 2.Comparison of Baseline Features Between the Included Set and the Excluded Set.**

| Variable | Included(n=561) | Excluded(n=618) | P Value |
| --- | --- | --- | --- |
| Baseline GDS-15 scores | 3.00(3.00,4.00) | 3.00(3.00,4.00) | 0.241 |
| Age at Enrollment | 62(55,69) | 64(58,70) | <0.001* |
| Education | 16(14,18) | 16(14,18) | 0.016* |
| Duration | 184(91,638) | 273(151, 579) | <0.001* |
| Follow-up time | 7(4,11) | 1(1,2) | <0.001* |
| State | OFF(561,100%) | OFF(80, 12.9%), ON(66, 10.6%), NA(472, 76.3%) |  |
| Sex |  |  | 1 |
| Female | 220(39.2%) | 242(39.2%) |  |
| Male | 341(60.8%) | 376(60.8%) |  |
| Handedness |  |  | 0.673 |
| Left | 51(9.1%) | 50(8.1%) |  |
| Right | 494(88.1%) | 552(89.6%) |  |
| Mixed | 16(2.9%) | 14(2.3%) |  |
| Depression |  |  | 0.006* |
| 0 | 421(75.0%) | 505(81.7%) |  |
| 1 | 140(25.0%) | 113(18.3%) |  |
| Depression medication |  |  | 0.113 |
| 0 | 461(82.2%) | 529(85.6%) |  |
| 1 | 100(17.8%) | 89(14.4%) |  |
| Symptom onset side |  |  | 0.063 |
| 1 | 250(44.6%) | 270(43.7%) |  |
| 2 | 291(51.9%) | 328(53.1%) |  |
| 3 | 20(3.6%) | 14(2.3%) |  |
| 4 | 0 | 6(1.0%) |  |
| Hoehn & Yahr stage |  |  | <0.001* |
| 0 | 1(0.2%) | 0 |  |
| 1 | 219(39.0%) | 166(26.9%) |  |
| 2 | 328(58.5%) | 431(70.0%) |  |
| 3 | 13(2.3%） | 19(3.1%) |  |
| MSEADL score | 90.00 (90.00, 100.00) | 95.00 (90.00, 100.00) | <0.001* |
| ESS score | 5.00 (3.00, 8.00) | 5.00 (3.00, 8.00) | 0.184 |
| RBDSQ score | 4.00 (2.00, 6.00) | 3.00 (2.00, 6.00) | 0.003* |
| SCOPA-AUT |  |  |  |
| Gastrointestinal score | 4.00 (2.00, 6.00) | 3.00 (2.00, 6.00) | 0.003* |
| Urinary score | 2.00 (1.00, 4.00) | 2.00 (1.00, 4.00) | 0.045* |
| Cardiovascular score | 4.00 (2.00, 6.00) | 4.00 (2.00, 7.00) | <0.001* |
| Thermoregulatory score | 0.00 (0.00, 1.00) | 0.00 (0.00, 1.00) | 0.081 |
| Pupillomotor score | 1.00 (0.00, 2.00) | 1.00 (0.00, 2.00) | 0.766 |
| Sexual score | 0.00 (0.00, 1.00) | 0.00 (0.00, 1.00) | 0.383 |
| QUIP-CS score | 2.00 (0.00, 5.00) | 2.00 (0.00, 5.00) | 0.905 |
| STAI score | 4.00 (0.00, 4.00) | 4.00 (1.00, 4.00) | 0.941 |
| BJLOT total raw score | 94.00 (88.00, 98.00) | 95.00 (90.00, 98.00) | 0.029* |
| HVLT-R |  |  |  |
| Total recall score | 46.00 (38.00, 53.00) | 46.00 (38.50, 54.00) | 0.471 |
| Delayed recall score | 45.00 (37.00, 54.00) | 47.00 (36.00, 56.00) | 0.224 |
| Retention score | 48.00 (39.00, 55.00) | 48.00 (39.00, 55.50) | 0.866 |
| Recognition-discrimination index score | 45.00 (37.00, 53.00) | 49.00 (39.00, 57.00) | 0.005* |
| LNS score | 10.00 (9.00, 12.00) | 10.00 (9.00, 12.00) | 0.867 |
| MSFT score | 51.00 (44.00, 57.00) | 51.50 (45.00, 58.00) | 0.125 |
| SDMT score | 42.00 (33.00, 48.00) | 42.00 (34.00, 49.00) | 0.175 |
| MDS-UPDRS I |  |  |  |
| Congitive impairment | 0.00 (0.00, 1.00) | 0.00 (0.00, 1.00) | 0.903 |
| Hallucinations and psychosis | 0.00 (0.00, 0.00) | 0.00 (0.00, 0.00) | 0.015* |
| Depressed moods | 0.00 (0.00, 1.00) | 0.00 (0.00, 1.00) | 0.700 |
| Anxious mood | 0.00 (0.00, 1.00) | 0.00 (0.00, 1.00) | 0.586 |
| Apathy | 0.00 (0.00, 0.00) | 0.00 (0.00, 0.00) | 0.971 |
| Features dopamine dysregulation syndrome | 0.00 (0.00, 0.00) | 0.00 (0.00, 0.00) | 0.269 |
| Sleep problems night | 1.00 (0.00, 2.00) | 1.00 (0.00, 2.00) | 0.426 |
| Daytime sleepiness | 1.00 (0.00, 1.00) | 1.00 (0.00, 1.00) | 0.667 |
| Pain and other sensations | 1.00 (0.00, 1.00) | 1.00 (0.00, 1.00) | 0.214 |
| Urinary problems | 0.00 (0.00, 1.00) | 1.00 (0.00, 1.00) | 0.007* |
| Constipation problems | 0.00 (0.00, 1.00) | 0.00 (0.00, 1.00) | 0.794 |
| Lightheadedness on standing | 0.00 (0.00, 1.00) | 0.00 (0.00, 1.00) | 0.716 |
| Fatigue | 1.00 (0.00, 1.00) | 1.00 (0.00, 1.00) | 0.762 |
| MDS-UPDRS II |  |  |  |
| Speech1 | 0.00 (0.00, 1.00) | 0.00 (0.00, 1.00) | 0.670 |
| Saliva drooling | 0.00 (0.00, 1.00) | 0.00 (0.00, 1.00) | 0.441 |
| Chewing and swallowing | 0.00 (0.00, 0.00) | 0.00 (0.00, 0.00) | 0.003* |
| Eating tasks | 0.00 (0.00, 1.00) | 0.00 (0.00, 1.00) | 0.010* |
| Dressing | 0.00 (0.00, 1.00) | 0.00 (0.00, 1.00) | 0.810 |
| Hygiene | 0.00 (0.00, 1.00) | 0.00 (0.00, 0.00) | 0.394 |
| Handwriting | 1.00 (0.00, 1.00) | 1.00 (0.00, 2.00) | 0.092 |
| Doing hobbies and other activities | 0.00 (0.00, 1.00) | 0.00 (0.00, 1.00) | 0.936 |
| Turning in bed | 0.00 (0.00, 1.00) | 0.00 (0.00, 1.00) | 0.359 |
| Tremor | 1.00 (1.00, 1.00) | 1.00 (1.00, 2.00) | 0.003* |
| Getting out of bed car or deep chair | 0.00 (0.00, 1.00) | 0.00 (0.00, 1.00) | 0.688 |
| Walking and balance | 0.00 (0.00, 1.00) | 0.00 (0.00, 1.00) | 0.777 |
| Freezing | 0.00 (0.00, 0.00) | 0.00 (0.00, 0.00) | 0.544 |
| MDS-UPDRS III |  |  |  |
| Speech2 | 0.00 (0.00, 1.00) | 0.00 (0.00, 1.00) | 0.729 |
| Facial expression | 1.00 (1.00, 2.00) | 1.00 (1.00, 2.00) | 0.778 |
| Rigidity | 3.00 (2.00, 5.00) | 3.00 (2.00, 6.00) | 0.086 |
| Finger tapping | 2.00 (1.00, 3.00) | 2.00 (1.00, 3.00) | <0.001* |
| Hand movements | 1.00 (1.00, 3.00) | 2.00 (1.00, 3.00) | <0.001* |
| Pronation supination | 1.00 (1.00, 2.00) | 2.00 (1.00, 3.00) | <0.001* |
| Toe tapping | 2.00 (1.00, 3.00) | 2.00 (1.00, 3.00) | 0.024* |
| Leg agility | 1.00 (0.00, 2.00) | 1.00 (0.00, 2.00) | 0.111 |
| Arising from chair | 0.00 (0.00, 0.00) | 0.00 (0.00, 0.00) | 0.403 |
| Gait | 1.00 (0.00, 1.00) | 1.00 (0.00, 1.00) | 0.004* |
| Freezing of gait | 0.00 (0.00, 0.00) | 0.00 (0.00, 0.00) | 0.036* |
| Postural stability | 0.00 (0.00, 0.00) | 0.00 (0.00, 0.00) | 0.564 |
| Posture | 1.00 (0.00, 1.00) | 1.00 (0.00, 1.00) | 0.776 |
| Global spontaneity of movement | 1.00 (1.00, 2.00) | 1.00 (1.00, 2.00) | 0.165 |
| Postural tremor | 0.00 (0.00, 1.00) | 1.00 (0.00, 1.00) | 0.151 |
| Kinetic tremor | 0.00 (0.00, 1.00) | 1.00 (0.00, 2.00) | 0.058 |
| Rest tremor amplitude | 1.00 (0.00, 2.00) | 2.00 (0.00, 3.00) | 0.001* |
| Constancy of rest tremor | 1.00 (0.00, 2.00) | 1.00 (0.00, 3.00) | 0.002* |

According to the screening process for PD patients, we removed the missing data before assessing the progression of depression. Therefore, the proportion of missing values was calculated based on 579 patients.

**Supplementary Table 3. Missing data distribution**

| Feature predictor | Missing data |
| --- | --- |
| Depression medication | 12 (2.07%) |
| HVLT-R |  |
| Recognition-discrimination index score | 3 (0.52%) |
| Total recall score | 1 (0.17%) |
| Delayed recall score | 1 (0.17%) |
| Retention score | 1 (0.17%) |
| LNS score | 2 (0.35%) |
| BJLOT total raw score | 2 (0.35%) |
| Handedness | 1 (0.17%) |
| SDMT score | 1 (0.17%) |

To further validate our findings, we conducted a sensitivity analysis using multiple imputation (MI). The results (Supplementary Table 4) show that the model remains robust across different data handling strategies. Notably, while the primary model yielded a solid C-index of 0.745-0.747, the MI-based model showed an even higher predictive performance (0.807-0.812). This comparison demonstrated that our findings were conservative and reliable, as the primary model still performed well despite the slight loss of power from exclusion. The selected top 20 variables captured the core predictors of PD progression effectively, as their performance was highly consistent with the full-variable models.

**Supplementary Table 4. Sensitivity Analysis of Model Performance: Comparison between Complete-Case Analysis and Multiple Imputation**

| C-index | RSF (excluding missing data) | RSF (multiple imputation) |
| --- | --- | --- |
| Models trained with all variables | | |
| Train set | 0.890 (95% CI: 0.889-0.891) | 0.897 (95% CI: 0.869-0.921) |
| Test set | 0.747 (95% CI: 0.744-0.749) | 0.807 (95% CI: 0.748-0.864) |
| Models trained with top 20 variables | | |
| Train set | 0.873 (95% CI: 0.872-0.874) | 0.884 (95% CI: 0.852-0.911) |
| Test set | 0.745 (95% CI: 0.742-0.748) | 0.812 (95% CI: 0.751-0.871) |

**Variables selected of XGBoost and SSVM**

Variables selected of XGBoost and SSVM via univariate analysis in the train set: Anxious mood, BJLOT total raw score, Baseline GDS-15 scores, Cardiovascular score, Congitive impairment, Constipation problems, Daytime sleepiness, Depressed moods, Doing hobbies and other activities, Education, Fatigue, Finger tapping, Gastrointestinal score, LNS score, Pain and other sensations, Pupillomotor score, RBDSQ score, SDMT score, Sleep problems night, Thermoregulatory score, Total recall score, Depression. There are 22 variables in total.

Variables selected of XGBoost and SSVM via LASSO analysis in the train set: BJLOT total raw score, Baseline GDS-15 scores, Constipation problems, Gastrointestinal score, SDMT score.

The variable selection process followed a pre-defined analytical pipeline rather than being pre-specified. To handle high-dimensional clinical data and improve the EPV ratio, univariate screening (P < 0.1) was conducted strictly within the training set to filter out noise. Furthermore, we implemented LASSO (Least Absolute Shrinkage and Selection Operator) regression as a sensitivity check for feature selection. The LASSO model independently identified five key predictors (BJLOT total raw score, Baseline GDS-15 scores, Constipation problems, Gastrointestinal score, and SDMT score). Notably, these variables were all captured within our primary univariate screening and SHAP-based selection, reinforcing the robustness and reliability of our feature selection process. This multi-step selection framework was designed to maximize model stability and ensure that the risk scoring system is driven by feature predictors rather than data-driven artifacts.

The reason why only XGBOOST and SSVM employed feature selection was that severe overfitting was observed when using all the features. After feature selection, the C-index of the test set remained low, indicating that these two models are not suitable for the prediction in this study

**Supplementary Table 5. Baseline Characteristics of the Non-progressors and Progressors Groups**

| Variable | All(n=496) | Non-progressors(n=402) | Progressors(n=94) | P Value |
| --- | --- | --- | --- | --- |
| Baseline GDS-15 scores | 3.00(3.00,4.00) | 3.00(3.00,3.00) | 4.00(3.00,4.75) | <0.001* |
| Age at Enrollment | 62(55,69) | 62(55,69) | 64(55,69) | 0.810 |
| Education | 16(14,18) | 16.00(14.00,18.00) | 16.00(13.00,18.00) | 0.226 |
| Duration | 184(91,638) | 184.00(91.00,638.75) | 228.00(92.00,630.25) | 0.565 |
| Follow-up time | 7.00(5.00,11.00) | 7.00(5.00,11.00) | 8.00(6.00,11.00) | 0.129 |
| Time to event | 7(4,11) | 7(5,11) | 4(2,6) | <0.001* |
| Sex |  |  |  | 0.907 |
| Female | 192(38.7%) | 155(38.6%) | 37(39.4%) |  |
| Male | 304(61.3%) | 24(61.4%) | 57(60.6%) |  |
| Handedness |  |  |  | 0.507 |
| Left | 48(9.7%) | 36(9.0%) | 12(12.8%) |  |
| Right | 434(87.5%) | 355(88.3%) | 79(84.0%) |  |
| Mixed | 14(2.8%) | 11(2.7%) | 3(3.2%) |  |
| Depression |  |  |  | 0.006* |
| 0 | 415(83.7%) | 322(80.1%) | 62(66.0%) |  |
| 1 | 81(16.3%) | 80(19.9%) | 32(34.0%) |  |
| Depression medication |  |  |  | 0.198 |
| 0 | 415(83.7%) | 341(84.8%) | 74(78.7%) |  |
| 1 | 81(16.3%) | 61(15.2%) | 20(21.3%) |  |
| Symptom onset side |  |  |  | 0.283 |
| Left | 220(44.4%) | 185(46.0%) | 35(37.2%) |  |
| Right | 260(52.4%) | 205(51.0%) | 55(58.5%) |  |
| Bilateral | 16(3.2%) | 12(3.0%) | 4(4.3%) |  |
| Hoehn & Yahr stage |  |  |  | 0.703 |
| 0 | 1(0.2%) | 1(0.2%) | 0 |  |
| 1 | 196(39.5%) | 162(40.3%) | 34(36.2%) |  |
| 2 | 287(57.9%) | 229(57.0%) | 58(61.7%) |  |
| 3 | 12(2.4%） | 10(2.5%) | 2(2.1%) |  |
| MSEADL score | 90.00(90.00,100.00) | 90.00(90.00,100.00) | 90.00(90.00,100.00) | 0.067 |
| ESS score | 5.00(3.00,8.00) | 5.00(3.00,8.00) | 6.00(4.00,9.00) | 0.030* |
| RBDSQ score | 4.00(2.00,6.00) | 4.00(2.00,6.00) | 5.00(3.25,7.00) | <0.001* |
| SCOPA-AUT |  |  |  |  |
| Gastrointestinal score | 2.00(0.00,4.00) | 2.00(0.00,3.00) | 3.00(1.00,5.75) | <0.001* |
| Urinary score | 4.00(2.00,6.00) | 4.00(2.00,5.00) | 4.00(3.00,7.00) | 0.005* |
| Cardiovascular score | 0.00(0.00,1.00) | 0.00(0.00,1.00) | 0.50(0.00,1.00) | 0.008* |
| Thermoregulatory score | 1.00(0.00,2.00) | 1.00(0.00,2.00) | 2.00(0.25,3.00) | <0.001* |
| Pupillomotor score | 0.00(0.00,1.00) | 0.00(0.00,1.00) | 0.00(0.00,1.00) | 0.112 |
| Sexual score | 2.00(0.00,5.00) | 2.00(0.00,5.00) | 2.00(0.00,5.00) | 0.775 |
| QUIP-CS score | 4.00(0.00,4.00) | 4.00(0.00,4.00) | 4.00(0.25,4.00) | 0.670 |
| STAI score | 94.00(88.00,98.00) | 94.00(88.00,97.00) | 95.00(89.00,99.00) | 0.238 |
| BJLOT total raw score | 13.00(11.00,14.00) | 13.00(12.00,14.00) | 12.00(10.00,14.00) | <0.001* |
| HVLT-R |  |  |  |  |
| Total recall score | 46.00(38.00,54.00) | 46.00(39.00,54.00) | 43.50(35.25,51.00) | 0.090 |
| Delayed recall score | 45.00(36.75,55.00) | 45.00(37.00,55.00) | 44.00(36.25,52.75) | 0.277 |
| Retention score | 48.00(39.00,55.00) | 48.00(39.00,55.00) | 48.00(39.00,56.00) | 0.614 |
| Recognition-discrimination index score | 47.00(37.00,53.25) | 47.00(38.00,54.00) | 45.00(36.25,53.00) | 0.280 |
| LNS score | 11.00(9.00,12.00) | 11.00(9.00,12.00) | 9.50(8.00,11.00) | 0.002* |
| MSFT score | 50.50(44.00,57.00) | 51.00(44.00,57.00) | 49.50(43.25,56.75) | 0.386 |
| SDMT score | 42.00(33.00,48.00) | 42.00(35.00,48.00) | 38.00(29.25,45.00) | 0.003* |
| MDS-UPDRS I |  |  |  |  |
| Congitive impairment | 0.00(0.00,1.00) | 0.00(0.00,1.00) | 0.00(0.00,1.00) | 0.218 |
| Hallucinations and psychosis | 0.00(0.00,0.00) | 0.00(0.00,0.00) | 0.00(0.00,0.00) | 0.038* |
| Depressed moods | 0.00(0.00,1.00) | 0.00(0.00,0.00) | 0.00(0.00,1.00) | <0.001* |
| Anxious mood | 0.00(0.00,1.00) | 0.00(0.00,1.00) | 0.00(0.00,1.00) | 0.018 |
| Apathy | 0.00(0.00,0.00) | 0.00(0.00,0.00) | 0.00(0.00,0.75) | 0.038 |
| Features dopamine dysregulation syndrome | 0.00(0.00,0.00) | 0.00(0.00,0.00) | 0.00(0.00,0.00) | 0.525 |
| Sleep problems night | 1.00(0.00,2.00) | 1.00(0.00,2.00) | 1.00(0.00,2.00) | 0.047* |
| Daytime sleepiness | 1.00(0.00,1.00) | 1.00(0.00,1.00) | 1.00(0.00,2.00) | 0.019* |
| Pain and other sensations | 1.00(0.00,1.00) | 0.50(0.00,1.00) | 1.00(0.00,2.00) | <0.001* |
| Urinary problems | 0.00(0.00,1.00) | 0.00(0.00,1.00) | 1.00(0.00,1.00) | 0.060 |
| Constipation problems | 0.00(0.00,1.00) | 0.00(0.00,1.00) | 0.50(0.00,1.00) | 0.001* |
| Lightheadedness on standing | 0.00(0.00,1.00) | 0.00(0.00,1.00) | 0.00(0.00,1.00) | 0.226 |
| Fatigue | 1.00(0.00,1.00) | 1.00(0.00,1.00) | 1.00(0.00,1.00) | 0.053 |
| MDS-UPDRS II |  |  |  |  |
| Speech1 | 0.00(0.00,1.00) | 0.00(0.00,1.00) | 0.00(0.00,1.00) | 0.435 |
| Saliva drooling | 0.00(0.00,1.00) | 0.00(0.00,1.00) | 0.00(0.00,2.00) | 0.158 |
| Chewing and swallowing | 0.00(0.00,0.00) | 0.00(0.00,0.00) | 0.00(0.00,0.00) | 0.237 |
| Eating tasks | 0.00(0.00,1.00) | 0.00(0.00,1.00) | 0.00(0.00,1.00) | 0.070 |
| Dressing | 0.00(0.00,1.00) | 0.00(0.00,1.00) | 0.00(0.00,1.00) | 0.090 |
| Hygiene | 0.00(0.00,1.00) | 0.00(0.00,0.75) | 0.00(0.00,1.00) | 0.262 |
| Handwriting | 1.00(0.00,1.00) | 1.00(0.00,1.00) | 1.00(0.00,2.00) | 0.161 |
| Doing hobbies and other activities | 0.00(0.00,1.00) | 0.00(0.00,1.00) | 1.00(0.00,1.00) | 0.012* |
| Turning in bed | 0.00(0.00,1.00) | 0.00(0.00,1.00) | 0.00(0.00,1.00) | 0.092 |
| Tremor | 1.00(1.00,1.00) | 1.00(1.00,1.00) | 1.00(1.00,1.00) | 0.204 |
| Getting out of bed car or deep chair | 0.00(0.00,1.00) | 0.00(0.00,1.00) | 0.50(0.00,1.00) | 0.102 |
| Walking and balance | 0.00(0.00,1.00) | 0.00(0.00,1.00) | 0.00(0.00,1.00) | 0.216 |
| Freezing | 0.00(0.00,0.00) | 0.00(0.00,0.00) | 0.00(0.00,0.00) | 0.528 |
| MDS-UPDRS III |  |  |  |  |
| Speech 2 | 0.00(0.00,1.00) | 0.00(0.00,1.00) | 0.00(0.00,1.00) | 0.579 |
| Facial expression | 1.00(1.00,2.00) | 1.00(1.00,2.00) | 1.00(1.00,1.00) | 0.864 |
| Rigidity | 3.00(2.00,5.00) | 3.00(2.00,5.00) | 3.00(1.25,5.00) | 0.594 |
| Finger tapping | 2.00(1.00,3.00) | 2.00(1.00,3.00) | 2.00(1.00,3.00) | 0.453 |
| Hand movements | 1.00(1.00,2.25) | 1.00(1.00,2.00) | 1.00(1.00,2.75) | 0.834 |
| Pronation supination | 1.00(1.00,2.00) | 1.00(1.00,2.00) | 1.00(1.00,2.00) | 0.336 |
| Toe tapping | 2.00(1.00,3.00) | 2.00(1.00,3.00) | 1.00(1.00,2.75) | 0.207 |
| Leg agility | 1.00(0.00,2.00) | 1.00(0.00,2.00) | 1.00(0.00,1.00) | 0.293 |
| Arising from chair | 0.00(0.00,0.00) | 0.00(0.00,0.00) | 0.00(0.00,0.00) | 0.507 |
| Gait | 1.00(0.00,1.00) | 1.00(0.00,1.00) | 1.00(0.00,1.00) | 0.585 |
| Freezing of gait | 0.00(0.00,0.00) | 0.00(0.00,0.00) | 0.00(0.00,0.00) | 0.463 |
| Postural stability | 0.00(0.00,0.00) | 0.00(0.00,0.00) | 0.00(0.00,0.00) | 0.882 |
| Posture | 1.00(0.00,1.00) | 1.00(0.00,1.00) | 1.00(0.00,1.00) | 0.971 |
| Global spontaneity of movement | 1.00(1.00,2.00) | 1.00(1.00,2.00) | 1.00(1.00,2.00) | 0.376 |
| Postural tremor | 0.00(0.00,1.00) | 0.50(0.00,1.00) | 0.00(0.00,1.00) | 0.817 |
| Kinetic tremor | 0.00(0.00,1.00) | 0.00(0.00,1.00) | 0.00(0.00,1.00) | 0.307 |
| Rest tremor amplitude | 1.00(0.00,2.00) | 1.00(0.00,2.00) | 1.00(0.00,2.00) | 0.973 |
| Constancy of rest tremor | 1.00(0.00,2.00) | 1.00(0.00,2.00) | 1.00(0.00,2.00) | 0.406 |

Values are n (%), mean ± SD, or median (Q1-Q3), unless otherwise indicated.MSEADL, Modified Schwab and England Activities of Daily Living; ESS, Epworth Sleepiness Scale; RBDSQ, Rapid Eye Movement Sleep Behavior Disorder Screening Questionnaire; SCOPA-AUT, Scales for Outcomes in Parkinson's Disease-Autonomic questionnaire; QUIP-CS, Questionnaire for Impulsive-Compulsive Disorders in Parkinson’s Disease–Current Short Version; STAI, State Trait Anxiety Total Score; BJLOT, Benton Judgment of Line Orientation Test; HVLT-R, Hopkins Verbal Learning Test–Revised; LNS, Letter-Number Sequencing Test; MSFT, Modified Semantic Fluency Test; SDMT, Symbol Digit Modalities Test; MDS-UPDRS, Movement Disorder Society Revision of the Unified Parkinson Disease Rating Scale.

**Supplementary Table 6.Comparison of Baseline Features Between the Training Set and the Test Set.**

| Variable | All(n=496) | Training set(n=347) | Testing set(n=149) | P Value |
| --- | --- | --- | --- | --- |
| Baseline GDS-15 scores | 3.00(3.00,4.00) | 3.00(3.00,4.00) | 3.00(3.00,4.00) | 0.942 |
| Age at Enrollment | 62(55,69) | 63(55,69) | 61(55,69) | 0.277 |
| Education | 16(14,18) | 16.00(14.00,18.00) | 16.00(13.00,18.00) | 0.742 |
| Duration | 183(91,639) | 183(91,639) | 183(91,639) | 0.647 |
| Follow-up time | 7(5,11) | 8(5,11) | 7(5,11) | 0.085 |
| Time to event | 7(4,11) | 7(4,11) | 6(4,10) | 0.170 |
| Sex |  |  |  | 0.920 |
| Female | 192(38.7%) | 135(38.9%) | 57(38.3%) |  |
| Male | 304(61.3%) | 212(61.1%) | 92(61.7%) |  |
| Handedness |  |  |  | 0.681 |
| Left | 48(9.7%) | 35(10.1%) | 13(8.7%) |  |
| Right | 434(87.5%) | 301(86.7%) | 133(89.3%) |  |
| Mixed | 14(2.8%) | 11(3.2%) | 3(2.0%) |  |
| Depression |  |  |  | 0.640 |
| 0 | 415(83.7%) | 271(78.1%) | 113(75.8%) |  |
| 1 | 81(16.3%) | 76(21.9%) | 36(24.2%) |  |
| Depression medication |  |  |  | 0.895 |
| 0 | 415(83.7%) | 291(83.9%) | 124(83.5%) |  |
| 1 | 81(16.3%) | 56(16.1%) | 25(16.8%) |  |
| Symptom onset side |  |  |  | 0.067 |
| 1 | 220(44.4%) | 145(41.8%) | 75(50.3%) |  |
| 2 | 260(52.4%) | 193(55.6%) | 67(45.0%) |  |
| 3 | 16(3.2%) | 9(2.6%) | 7(4.7%) |  |
| Hoehn & Yahr stage |  |  |  | 0.900 |
| 0 | 1(0.2%) | 1(0.3%) | 0 |  |
| 1 | 196(39.5%) | 137(39.5%) | 59(39.6%) |  |
| 2 | 287(57.9%) | 200(57.6%) | 87(58.4%) |  |
| 3 | 12(2.4%） | 9(2.6%) | 3(2.0%) |  |
| MSEADL score | 90.00(90.00,100.00) | 90.00(90.00,100.00) | 90.00(90.00,100.00) | 0.799 |
| ESS score | 5.00(3.00,8.00) | 5.00(3.00,8.00) | 5.00(3.00,8.00) | 0.770 |
| RBDSQ score | 4.00(2.00,6.00) | 4.00(2.00,6.00) | 4.00(2.00,6.00) | 0.498 |
| SCOPA-AUT |  |  |  |  |
| Gastrointestinal score | 2.00(0.00,4.00) | 2.00(0.00,4.00) | 2.00(1.00,4.00) | 0.542 |
| Urinary score | 4.00(2.00,6.00) | 3.00(2.00,5.50) | 4.00(3.00,6.00) | 0.005* |
| Cardiovascular score | 0.00(0.00,1.00) | 0.00(0.00,1.00) | 0.00(0.00,1.00) | 0.662 |
| Thermoregulatory score | 1.00(0.00,2.00) | 1.00(0.00,2.00) | 1.00(0.00,2.00) | 0.113 |
| Pupillomotor score | 0.00(0.00,1.00) | 0.00(0.00,1.00) | 0.00(0.00,1.00) | 0.521 |
| Sexual score | 2.00(0.00,5.00) | 2.00(0.00,5.00) | 2.00(0.00,5.00) | 0.737 |
| QUIP-CS score | 4.00(0.00,4.00) | 4.00(0.00,4.00) | 4.00(0.00,4.00) | 0.506 |
| STAI score | 94.00(88.00,98.00) | 94.00(88.50,98.00) | 94.00(88.00,98.00) | 0.894 |
| BJLOT total raw score | 13.00(11.00,14.00) | 13.00(11.00,14.00) | 13.00(11.00,14.00) | 0.359 |
| HVLT-R |  |  |  |  |
| Total recall score | 46.00(38.00,54.00) | 46.00(39.00,54.00) | 45.00(38.00,52.00) | 0.249 |
| Delayed recall score | 45.00(36.75,55.00) | 45.00(37.00,53.00) | 44.00(34.00,55.00) | 0.193 |
| Retention score | 48.00(39.00,55.00) | 48.00(41.00,55.00) | 48.00(35.00,55.00) | 0.048* |
| Recognition-discrimination index score | 47.00(37.00,53.25) | 47.00(38.00,54.00) | 45.00(37.00,53.00) | 0.220 |
| LNS score | 11.00(9.00,12.00) | 11.00(9.00,12.00) | 11.00(9.00,12.00) | 0.653 |
| MSFT score | 50.50(44.00,57.00) | 51.00(45.00,57.00) | 49.00(42.00,55.00) | 0.026* |
| SDMT score | 42.00(33.00,48.00) | 42.00(33.00,48.00) | 42.00(35.00,48.00) | 0.556 |
| MDS-UPDRS I |  |  |  |  |
| Congitive impairment | 0.00(0.00,1.00) | 0.00(0.00,1.00) | 0.00(0.00,1.00) | 0.192 |
| Hallucinations and psychosis | 0.00(0.00,0.00) | 0.00(0.00,0.00) | 0.00(0.00,0.00) | 0.037* |
| Depressed moods | 0.00(0.00,1.00) | 0.00(0.00,0.50) | 0.00(0.00,1.00) | 0.305 |
| Anxious mood | 0.00(0.00,1.00) | 0.00(0.00,1.00) | 0.00(0.00,1.00) | 0.538 |
| Apathy | 0.00(0.00,0.00) | 0.00(0.00,0.00) | 0.00(0.00,0.00) | 0.350 |
| Features dopamine dysregulation syndrome | 0.00(0.00,0.00) | 0.00(0.00,0.00) | 0.00(0.00,0.00) | 0.507 |
| Sleep problems night | 1.00(0.00,2.00) | 1.00(0.00,2.00) | 1.00(0.00,2.00) | 0.266 |
| Daytime sleepiness | 1.00(0.00,1.00) | 1.00(0.00,1.00) | 1.00(0.00,1.00) | 0.386 |
| Pain and other sensations | 1.00(0.00,1.00) | 1.00(0.00,1.00) | 0.00(0.00,1.00) | 0.484 |
| Urinary problems | 0.00(0.00,1.00) | 0.00(0.00,1.00) | 1.00(0.00,1.00) | 0.124 |
| Constipation problems | 0.00(0.00,1.00) | 0.00(0.00,1.00) | 0.00(0.00,1.00) | 0.526 |
| Lightheadedness on standing | 0.00(0.00,1.00) | 0.00(0.00,1.00) | 0.00(0.00,1.00) | 0.944 |
| Fatigue | 1.00(0.00,1.00) | 1.00(0.00,1.00) | 1.00(0.00,1.00) | 0.529 |
| MDS-UPDRS II |  |  |  |  |
| Speech1 | 0.00(0.00,1.00) | 0.00(0.00,1.00) | 0.00(0.00,1.00) | 0.193 |
| Saliva drooling | 0.00(0.00,1.00) | 0.00(0.00,1.00) | 0.00(0.00,1.00) | 0.950 |
| Chewing and swallowing | 0.00(0.00,0.00) | 0.00(0.00,0.00) | 0.00(0.00,0.00) | 0.054 |
| Eating tasks | 0.00(0.00,1.00) | 0.00(0.00,1.00) | 0.00(0.00,1.00) | 0.975 |
| Dressing | 0.00(0.00,1.00) | 0.00(0.00,1.00) | 0.00(0.00,1.00) | 0.618 |
| Hygiene | 0.00(0.00,1.00) | 0.00(0.00,0.00) | 0.00(0.00,1.00) | 0.214 |
| Handwriting | 1.00(0.00,1.00) | 1.00(0.00,1.00) | 1.00(0.00,2.00) | 0.618 |
| Doing hobbies and other activities | 0.00(0.00,1.00) | 0.00(0.00,1.00) | 0.00(0.00,1.00) | 0.733 |
| Turning in bed | 0.00(0.00,1.00) | 0.00(0.00,1.00) | 0.00(0.00,1.00) | 0.554 |
| Tremor | 1.00(1.00,1.00) | 1.00(1.00,1.00) | 1.00(1.00,1.00) | 0.958 |
| Getting out of bed car or deep chair | 0.00(0.00,1.00) | 0.00(0.00,1.00) | 0.00(0.00,1.00) | 0.806 |
| Walking and balance | 0.00(0.00,1.00) | 0.00(0.00,1.00) | 0.00(0.00,1.00) | 0.776 |
| Freezing | 0.00(0.00,0.00) | 0.00(0.00,0.00) | 0.00(0.00,0.00) | 0.378 |
| MDS-UPDRS III |  |  |  |  |
| Speech2 | 0.00(0.00,1.00) | 0.00(0.00,1.00) | 0.00(0.00,1.00) | 0.436 |
| Facial expression | 1.00(1.00,2.00) | 1.00(1.00,2.00) | 1.00(1.00,1.00) | 0.788 |
| Rigidity | 3.00(2.00,5.00) | 3.00(2.00,5.00) | 3.00(1.00,5.00) | 0.314 |
| Finger tapping | 2.00(1.00,3.00) | 2.00(1.00,3.00) | 2.00(1.00,3.00) | 0.149 |
| Hand movements | 1.00(1.00,2.25) | 1.00(1.00,2.00) | 1.00(1.00,3.00) | 0.924 |
| Pronation supination | 1.00(1.00,2.00) | 1.00(1.00,2.00) | 1.00(1.00,3.00) | 0.383 |
| Toe tapping | 2.00(1.00,3.00) | 2.00(1.00,3.00) | 1.00(1.00,3.00) | 0.845 |
| Leg agility | 1.00(0.00,2.00) | 1.00(0.00,2.00) | 1.00(0.00,2.00) | 0.582 |
| Arising from chair | 0.00(0.00,0.00) | 0.00(0.00,0.00) | 0.00(0.00,0.00) | 0.238 |
| Gait | 1.00(0.00,1.00) | 1.00(0.00,1.00) | 1.00(0.00,1.00) | 0.831 |
| Freezing of gait | 0.00(0.00,0.00) | 0.00(0.00,0.00) | 0.00(0.00,0.00) | 0.99 |
| Postural stability | 0.00(0.00,0.00) | 0.00(0.00,0.00) | 0.00(0.00,0.00) | 0.871 |
| Posture | 1.00(0.00,1.00) | 1.00(0.00,1.00) | 1.00(0.00,1.00) | 0.900 |
| Global spontaneity of movement | 1.00(1.00,2.00) | 1.00(1.00,2.00) | 1.00(1.00,2.00) | 0.952 |
| Postural tremor | 0.00(0.00,1.00) | 1.00(0.00,1.00) | 0.00(0.00,1.00) | 0.008* |
| Kinetic tremor | 0.00(0.00,1.00) | 0.00(0.00,1.00) | 0.00(0.00,1.00) | 0.523 |
| Rest tremor amplitude | 1.00(0.00,2.00) | 1.00(0.00,2.00) | 1.00(0.00,2.00) | 0.069 |
| Constancy of rest tremor | 1.00(0.00,2.00) | 1.00(0.00,2.00) | 1.00(0.00,2.00) | 0.329 |

Values are n (%), mean ± SD, or median (Q1-Q3), unless otherwise indicated.MSEADL, Modified Schwab and England Activities of Daily Living; ESS, Epworth Sleepiness Scale; RBDSQ, Rapid Eye Movement Sleep Behavior Disorder Screening Questionnaire; SCOPA-AUT, Scales for Outcomes in Parkinson's Disease-Autonomic questionnaire; QUIP-CS, Questionnaire for Impulsive-Compulsive Disorders in Parkinson’s Disease–Current Short Version; STAI, State Trait Anxiety Total Score; BJLOT, Benton Judgment of Line Orientation Test; HVLT-R, Hopkins Verbal Learning Test–Revised; LNS, Letter-Number Sequencing Test; MSFT, Modified Semantic Fluency Test; SDMT, Symbol Digit Modalities Test; MDS-UPDRS, Movement Disorder Society Revision of the Unified Parkinson Disease Rating Scale.

**Supplementary Table 7. Hyperparameter space of the models**

| Model | Parameter | Parameter Space |
| --- | --- | --- |
| XGBoost | Max depth | 3 to 10 |
|  | Learning rate | 0.01 to 0.2 |
|  | Subsample | 0.6 to 1.0 |
|  | Colsample bytree | 0.6 to 1.0 |
|  | Reg alpha | 0.0 to 1.0 |
|  | Reg lambda | 0.0 to 1.0 |
|  | Min child weight | 1 to 20 |
|  | Gamma | 0 to 0.5 |
| RSF | N estimators | 100 to 500 |
|  | Max depth | 3 to 15 |
|  | Min samples split | 2 to 50 |
|  | Min samples leaf | 1 to 20 |
|  | Max features | 0.1 to 0.9 |
| SSVM | α | 0.0001 to 1000 |
|  | Tol | 1e-7 to1e-3 |
|  | Max iter | 500 to 5000 |
| GBSA | N estimators | 150 to 350 |
|  | Learning rate | 0.01 to 0.15 |
|  | Max depth | 2 to 6 |
|  | Min samples split | 20 to 150 |
|  | Min samples leaf | 10 to 60 |
|  | Subsample | 0.6 to 1.0 |

**Supplementary Table 8. Hyperparameter of the models**

| Model | Parameter | Parameter Space |
| --- | --- | --- |
| XGBoost | Max depth | 3 |
|  | Learning rate | 0.0410 |
|  | Subsample | 0.7364 |
|  | Colsample bytree | 0.9118 |
|  | Reg alpha | 0.1294 |
|  | Reg lambda | 0.3365 |
|  | Min child weight | 4 |
|  | Gamma | 0.2003 |
| RSF | N estimators | 175 |
|  | Max depth | 6 |
|  | Min samples split | 36 |
|  | Min samples leaf | 19 |
|  | Max features | 0.4298 |
| SSVM | α | 0.0001 |
|  | Tol | 2.40e-05 |
|  | Max iter | 3916 |
| GBSA | N estimators | 179 |
|  | Learning rate | 0.0113 |
|  | Max depth | 3 |
|  | Min samples split | 32 |
|  | Min samples leaf | 25 |
|  | Subsample | 0.9539 |

**Calibration assessment of RSF model in the test set**

The calibration of the RSF model was evaluated to ensure the agreement between predicted probabilities and observed depressive progression. The time-dependent calibration curves for 2, 4, 6, 8, and 10-year follow-ups demonstrated excellent predictive accuracy (Fig.S1). Visually, the smoothing lines closely followed the 45-degree ideal line, particularly in the low-to-moderate risk deciles where the majority of the patient population was concentrated.

The Brier scores remained consistently low across all time points, ranging from 0.048 at 2 years to 0.152 at 10 years, all well below the commonly accepted threshold of 0.25. Specifically, for short-term outcomes (2 and 4 years), the calibration curves closely followed the 45-degree ideal line, with ECE values of 0.033 and 0.055, respectively. Regarding the mid-to-long-term predictions (6 to 10 years), despite slight fluctuations at higher probability ranges in the 6-year plot, the model regained strong calibration at the 10-year mark (ECE = 0.065).


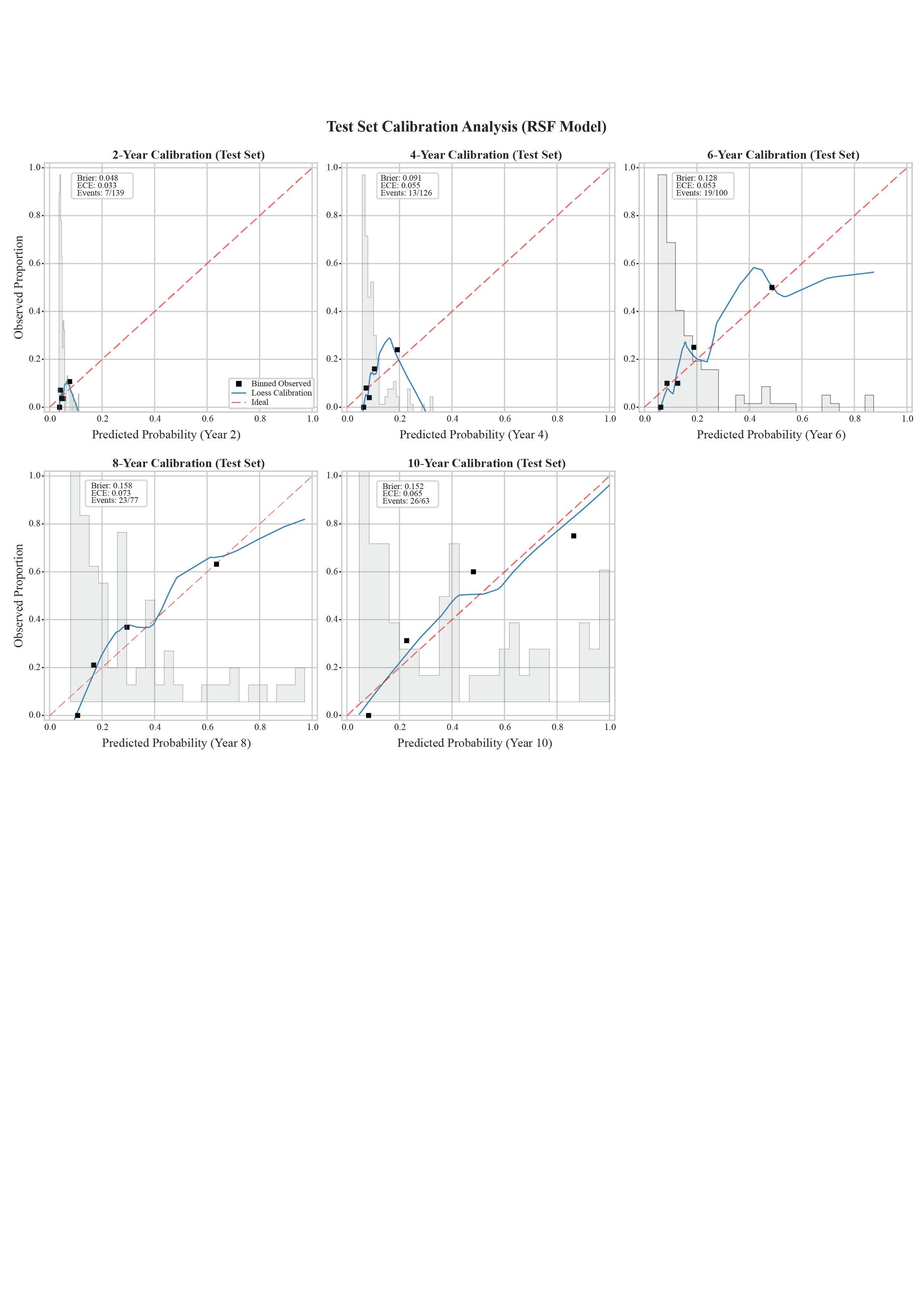


Fig. S1 Test set calibration analysis (RSF model)

**Decision Curve Analysis of RSF model in the test set**

To evaluate the clinical practicalities of the RSF model across various follow-up periods (2, 4, 6, 8, and 10 years), Decision Curve Analysis (DCA) was performed on the test set. As illustrated in Figure 6, the model demonstrated superior clinical net benefit compared to the "treat-all" or "treat-none" strategies across most threshold probabilities.

Short-term (2-4 years): Given the relatively low event rate (5.0%–10.3%), the clinical utility was observed within a narrower threshold range (0.06 - 0.16 at 4 years).

Long-term (6-10 years): As the cumulative incidence of dPD increased, the model’s utility expanded significantly. At 10 years, the model remained the optimal strategy across a threshold range of 0.05 to 0.99, achieving a net benefit of 0.3686 and 0.3214 at the 10% and 20% thresholds, respectively.

These findings indicate that the RSF model is a robust tool for clinical decision-making, particularly for identifying high-risk patients who would benefit from early, targeted interventions over a long-term horizon.


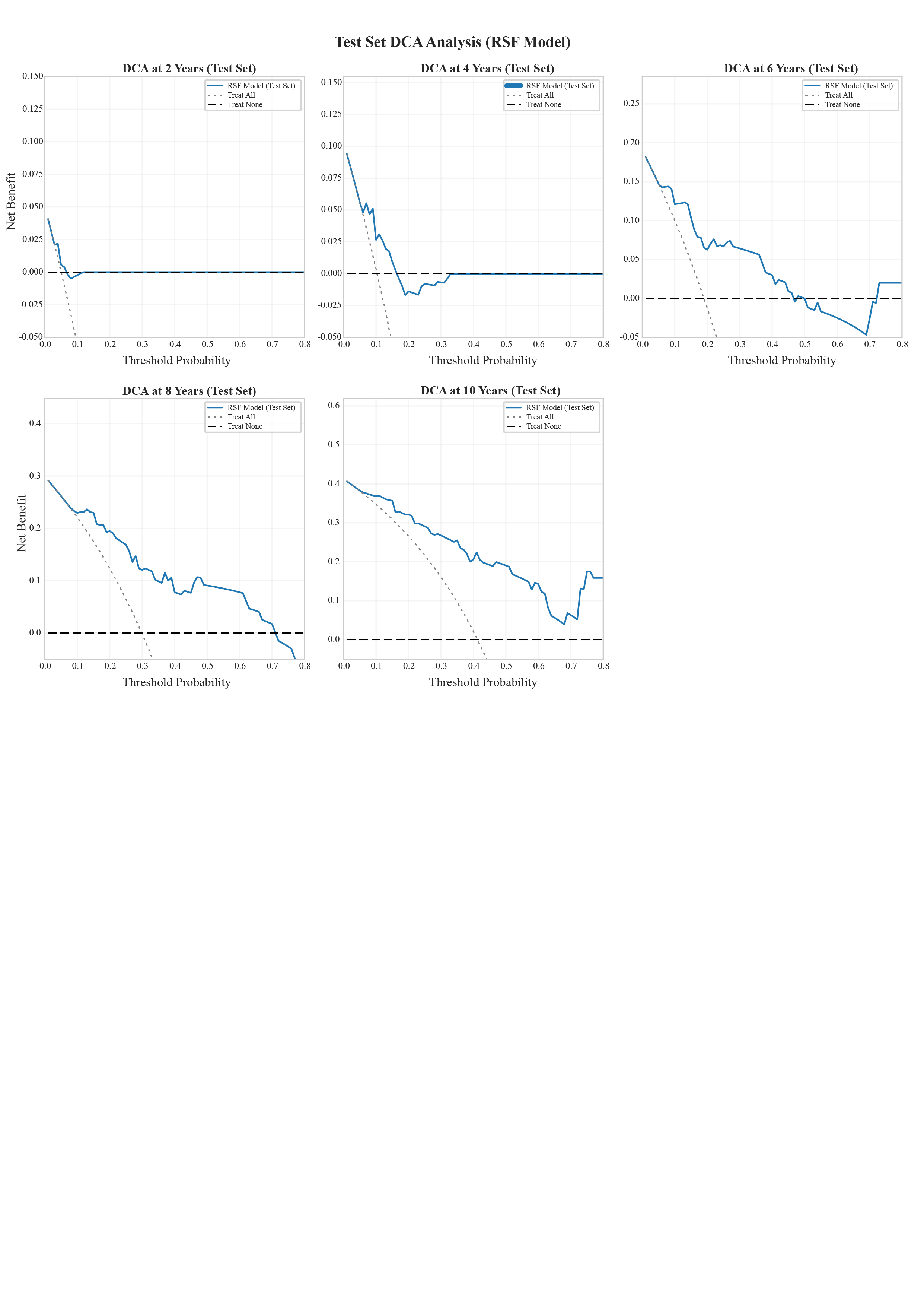


Fig. S2 Test set DCA analysis (RSF model)

**Supplementary Table 9. Sensitivity Analysis of Model Performance: Comparison between Complete-Case Analysis and Multiple Imputation**

| Time Point | Sample (N) | Event Rate (%) | Superior Range | NB (Pt = 0.10) | NB (Pt = 0.20) |
| --- | --- | --- | --- | --- | --- |
| 2 | 139 | 5.00% | 0.04 - 0.06 | -0.0024 | 0 |
| 4 | 126 | 10.30% | 0.06 - 0.16 | 0.0265 | -0.0139 |
| 6 | 100 | 19.00% | 0.06 - 0.87 | 0.1211 | 0.0625 |
| 8 | 77 | 29.90% | 0.08 - 0.97 | 0.2294 | 0.1948 |
| 10 | 63 | 41.30% | 0.05 - 0.99 | 0.3686 | 0.3214 |

**Sensitivity analysis for competing risks**

To assess the potential impact of competing risks (DBS surgery and loss to follow-up) on our findings, we compared the Aalen-Johansen (AJ) cumulative incidence function (CIF) with the standard Kaplan-Meier (KM) estimates. The AJ model yielded a cumulative incidence of 20.28% (95% CI: 16.33%–24.54%) at 10 years, compared to the KM estimate of 21.76%. The bias remained minimal throughout the follow-up period, ranging from 0.0002 at 2 years to 0.0269 at 12 years. The close alignment between the AJ CIF and KM curves (Fig.S3) confirmed that competing events did not substantially inflate the reported incidence of depressive progression, thereby reinforcing the stability of our risk stratification system.


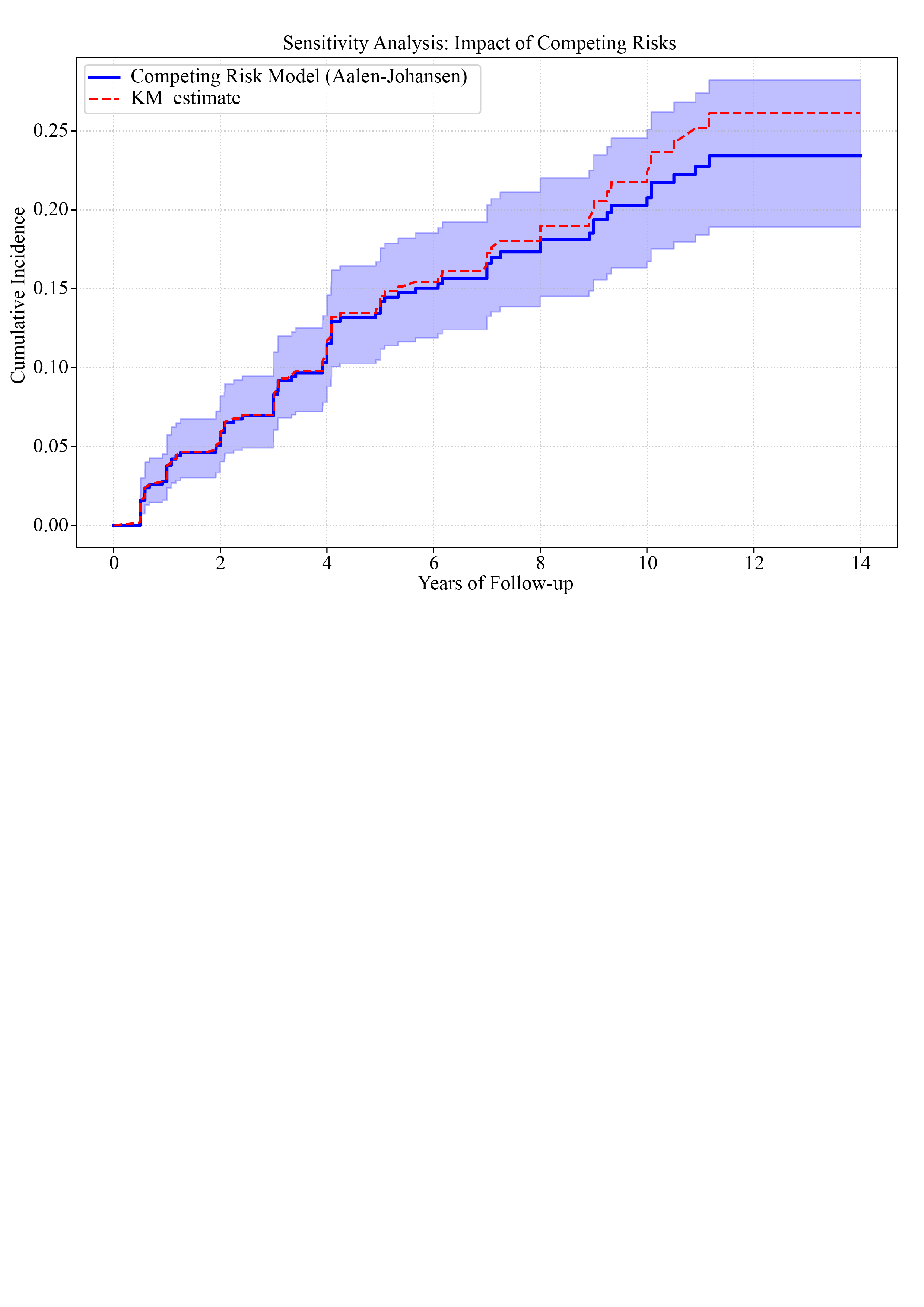


Fig. S3 Sensitivity analysis: impact of competing risks

**Supplementary Table 10. Comparison of Aalen-Johansen Estimation of Cumulative Incidence Rate of Events under Competing Risks Analysis with Kaplan-Meier Estimation.**

| Follow-up | AJ CIF (95% CI) | KM Estimate | Bias (KM-AJ) |
| --- | --- | --- | --- |
| 2 years | 0.0526 (0.0353 - 0.0748) | 0.0528 | 0.0002 |
| 4 years | 0.1035 (0.0781 - 0.1330) | 0.1051 | 0.0016 |
| 6 years | 0.1503 (0.1189 - 0.1852) | 0.1546 | 0.0043 |
| 8 years | 0.1734 (0.1387 - 0.2113) | 0.1806 | 0.0072 |
| 10 years | 0.2028 (0.1633 - 0.2454) | 0.2176 | 0.0148 |
| 12 years | 0.2343 (0.1892 - 0.2822) | 0.2612 | 0.0269 |
